# Tuning Perovskite Nanocrystal Synthesis via Amphiphilic Block Copolymer Templates and Solvent Interactions

**DOI:** 10.1021/acsami.4c13822

**Published:** 2024-10-30

**Authors:** Ya-Sen Sun, Kuan-Wei Wu, Orion Shih

**Affiliations:** † Department of Chemical Engineering, National Cheng Kung University, Tainan 701, Taiwan; ‡ National Synchrotron Radiation Research Center, Hsinchu 30076, Taiwan

**Keywords:** soft colloidal template, block copolymer, hierarchical emulsion, perovskite quantum dot, nucleation and growth, PL performance

## Abstract

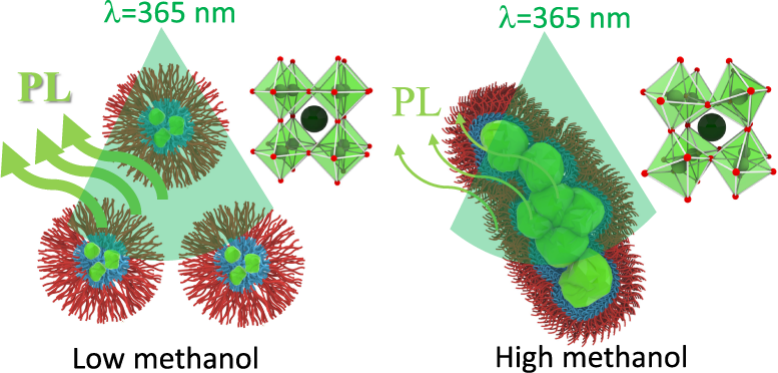

Amphiphilic block copolymer (a-BCP) micelles offer morphological diversity and dimensional tunability, making them suitable for the fabrication of perovskite nanocrystals. However, precise control over the nucleation and growth of perovskite nanocrystals using a-BCP colloidal templates remains underexplored. This study investigates the effects of toluene, methanol, and polystyrene-*block*-poly(2-vinylpyridine) (PS-*b*-P2VP) on the formation of cesium lead bromide (CsPbBr_3_) nanocrystals. The process involves four stages: (i) PS-*b*-P2VP micellization, (ii) PbBr_2_ complexation, (iii) coordination interaction with P2VP, and (iv) burst nucleation of CsPbBr_3_ nanocrystals. Toluene, a good solvent for PS but a nonsolvent for P2VP, PbBr_2_, and CsBr, facilitates the formation of PS-*b*-P2VP spherical micelles. Adding PbBr_2_ to these micelles in toluene results in multiple emulsion, dispersing PbBr_2_ microstructures (microemulsion) and forming [PbBr_3_]^−^ complexes encapsulated by the micelles (nanoemulsion). Prolonged stirring enhances this nanoemulsion. CsBr, insoluble in toluene, must be dissolved in methanol before being mixed with micelle-encapsulated complexes, promoting quick crystal nucleation. However, excess methanol weakens micellization, leading to the formation of fused micelles and irregular nanocrystals. At a high methanol content, [PbBr_4_]^2–^ complexes also form, driving CsPbBr_3_ to CsPb_2_Br_5_ transformation via Ostwald ripening, resulting in large CsPb_2_Br_5_ microcrystals that precipitate due to gravitational forces overcoming Brownian motion, destabilizing their dispersion in the solution.

## Introduction

Organic–inorganic (or all inorganic) lead halide perovskite nanocrystals (LHPNCs) have received much attention due to their impressive optical properties and cost-effective solution processing.^1,2^ These LHPNCs are notable for their narrow-band emission, high photoluminescence quantum yields, superior color purity, and adjustable composition and band gap. Additionally, they exhibit remarkable tolerance to structural defects, making them ideal for several photonic and optoelectronic applications. Nevertheless, Ahmed et al.^2^ indicates that despite their exceptional benefits, LHPNCs face significant environmental long-term stability challenges that impede their practical applications and commercialization. This instability is due to several factors like weak ionic bonds, low structural energy, loss of surface ligands, fragile ligand connections, separation of halide ions, low melting temperatures, and phase changes and polymorphism,^2^ to name a few. These problems reduce light emission and lower electron mobility. Another key challenge in the development of these LHPNCs is the ability to precisely control their shape with monodisperse size distribution, as the morphology significantly influences their optical, photonic, electronic, and optoelectronic properties.^3,4^

Sun et al.^4^ demonstrated that a ligand-mediated reprecipitation strategy at room temperature enables synthesis of LHPNCs with well-defined morphologies, dimensional tunability, and narrow size distribution. The basic concepts are based on soft colloidal templates through surface absorption and self-assembly of ligands to form core–shell perovskites, in which ligands (shells) absorb on the surface of perovskites (cores) and act as a capping layer to avoid moisture and oxygen.^1−4^

To act as a capping layer, ligands should have an amphiphilic property and weak interactions with the surface of perovskites. The amphiphilic property allows the formation and good dispersion of perovskites in organic solutions. Surfactants^5^ and copolymers^6^ are potential candidates to be used as ligands for mediating the synthesis of perovskites. Interactions between ligands and precursor salts should not be strong because strong interactions disturb and even prohibit the nucleation and growth of perovskites. Small ligands have been frequently used to mediate the formation of perovskites.^7^ Nevertheless, because of small sizes and highly dynamics,^8^ loss of surface ligands and fragile ligand connections may occur to cause a loss of halide ions from crystals and phase instability via Ostwald ripening. Additionally, the barrier imparted by small ligands may be too short to completely avoid degradation by moisture and oxygen. Moreover, small ligands may mediate the transformation of cesium lead bromide perovskite nanocrystals to other crystal forms.^9,10^ To address these problems, amphiphilic block copolymers^11−23^ or homopolymers^24−28^ become an alternative material to replace small molecule ligands.

Amphiphilic diblock copolymers (a-BCP) form core–shell micelles in a selective solvent, which selectively likes one block but dislikes the other block.^29^ As a result, solvophilic blocks form shells, and solvophobic blocks form cores. Morphologies of amphiphilic block copolymers in solution are versatile and diverse. A series of morphologies can be obtained by control over solvent quality, polymer concentration, volume fractions of constituent blocks, additives, and composition of cosolvents.^29^ Because of the morphological diversity and dimensional tunability in solution, a-BCP micelles have been employed as templates to fabricate inorganic nanomaterials, including nanostructured metals, metal oxides, porous carbons, semiconductors, and perovskite crystals.^1,11−23,30−45^

a-BCP materials bring several benefits to perovskite crystals. Long chains can offer longer resistance to the diffusion of moisture and oxygen than small ligands.^11,21^ Furthermore, long chains are expected to have much slower dynamics in comparison to small ligands. Nevertheless, highly dynamic ligand binding has not been tested to see whether it occurs in copolymer-templated synthesis of perovskites.

Hou et al. first employed polystyrene-*block*-poly(2-vinylpyridine) (PS-*b*-P2VP) amphiphilic diblock copolymer (a-BCP) core–shell micelles to successfully synthesize encapsulated CsPbBr_3_ nanocrystals dispersed in toluene, which is a nonsolvent for P2VP and CsPbBr_3_.^11^ Since the first discovery, many researchers have used PS-*b*-P2VP or other block copolymers as templates to mediate the formation of perovskite quantum dots.^1,12−23^

Nevertheless, several issues have not been completely addressed, including BCP micellization, hierarchical emulsion, salt complexation, coordinative interactions, crystallization behavior, and crystal instability for the hybrids of PbBr_2_ and CsBr in a nonsolvent added with PS-*b*-P2VP micelles. Furthermore, ligand-assisted reprecipitation (LARP) of CsPbBr_3_ nanocrystals frequently relies on two solvents with different solvent quality. In LARP synthesis, first, precursor salts and ligands are dissolved in a polar solvent to form transparent precursor solutions, followed by injection of a poor solvent to precipitate perovskite nanocrystals.^3,4^ To the best of our knowledge, effects of solvent quality on those issues have not yet been completely addressed yet. Particularly, the coexistence of two different solvents may also mediate BCP micellization.

This study aims to understand how soft BCP colloidal templates mediate the formation of perovskite crystals with high optical properties [photoluminescence (PL), photoluminescence quantum yield (PLQY), and time-resolved decay] and long-term instability. In this study, we used PS-*b*-P2VP as long ligands, for which lone pairs of electrons in pyridine groups selectively bind with Pb^2+^ ions^11^ and PS blocks act as capping layers to stabilize the dispersion of salt complexes and perovskite crystals in solutions. Two solvents were used, toluene and methanol. Toluene is a good solvent for PS blocks and methanol is a good solvent for P2VP blocks, PbBr_2_ and CsBr.

This study is outlined as follows. First, we studied hierarchical emulsions, BCP micellization, and salt complexation for PbBr_2_ in toluene added with PS-*b*-P2VP micelles before and after centrifugation. PbBr_2_ microcrystals were removed by centrifugation, and only salt complexes encapsulated by micelles were left in the precursor solutions. At this stage, CsBr was separately dissolved in methanol to prepare a CsBr solution because of its low solubility in toluene. Second, we studied optical properties and structural details of encapsulated CsPbBr_3_ nanoparticles after various contents of CsBr were added into centrifuged precursor solutions. Third, we studied the long-term instability of CsPbBr_3_ nanocrystals in mixed solutions. Finally, we correlated optical properties with the evolution of crystal lattices and nanoparticles.

## Experiment

### Materials

A polystyrene-*block*-poly(2-vinylpyridine) block copolymer [PS-*b*-P2VP: *M*_n_^PS^ = 49 kg/mol, *M*_n_^P2VP^ = 75 kg/mol, and *Đ* = 1.03] was purchased from Polymer Source, Inc. Lead bromide (PbBr_2_) and cesium bromide (CsBr) were purchased from Sigma-Aldrich. Toluene and methanol were purchased from UniRegion Bio-Tech and Honeywell, respectively. No purification was further performed on the chemicals. All of the chemicals were directly used as received.

### Nanocrystal Synthesis

**Scheme 1:**
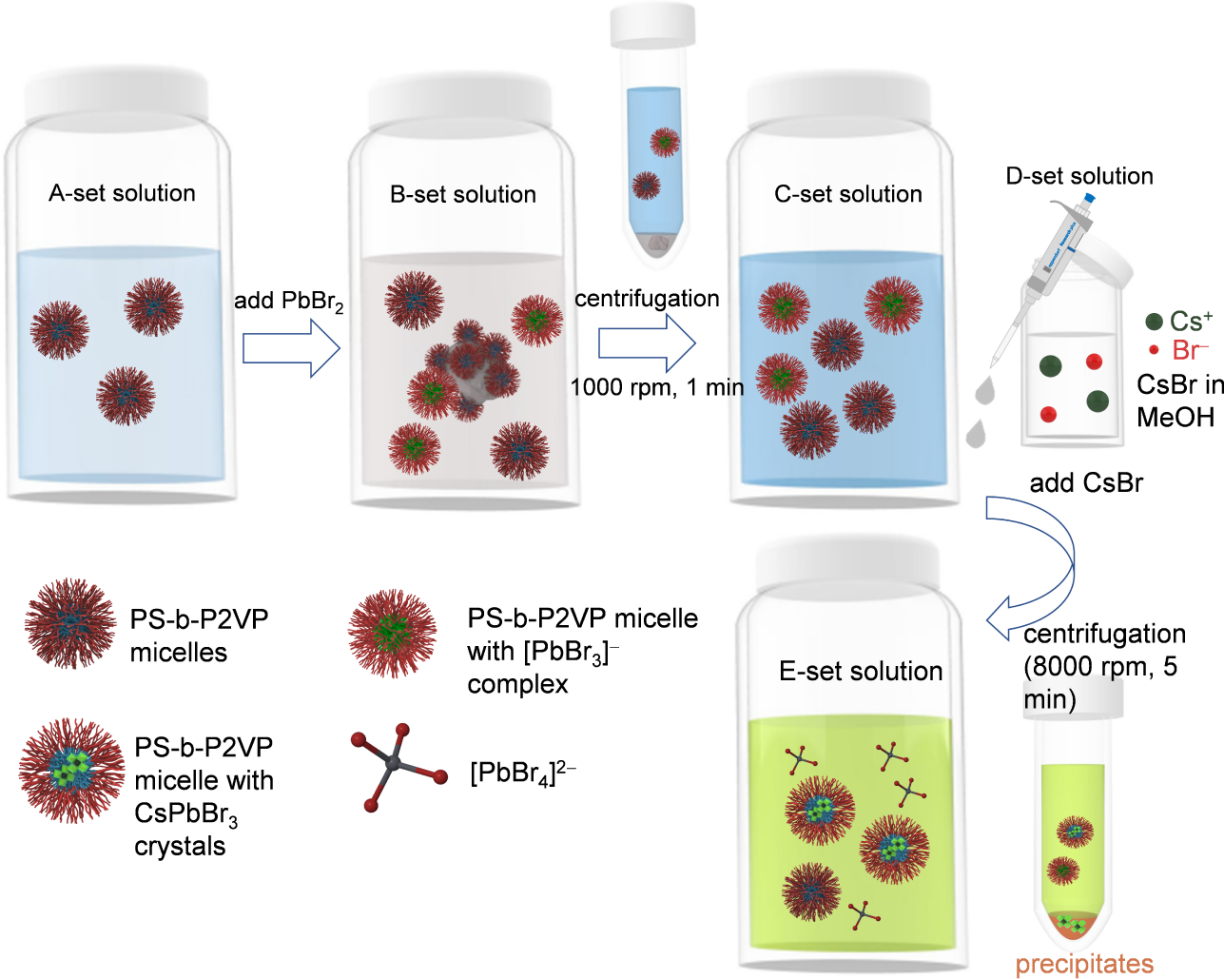
Synthesis Procedure for the CsPbBr_3_ Nanocrystals Prepared by the Templating of PS-*b*-P2VP Core–Shell Micelles

25 mg of PS-*b*-P2VP powders were dissolved in 5 mL of toluene to prepare PS-*b*-P2VP solutions with a mass concentration of 5 mg/mL, corresponding to a mass percentage concentration of ∼0.5 wt %. The PS-*b*-P2VP solutions were sonicated at 40 °C for 1 h. After sonication, the solutions appeared to be bluish. This color is due to Rayleigh scattering,^46,47^ indicating that the PS-*b*-P2VP formed micelles in toluene. Although the exact critical micellization concentration (CMC) of the PS-*b*-P2VP is unavailable, prior work^47^ on a similar BCP shows micelle formation at concentrations between 0.1 and 1 wt % in toluene, suggesting a CMC below 0.1 wt %. In this study, the BCP concentration (∼0.5 wt %) was above the CMC, ensuring effective micelle formation. For brevity, PS-*b*-P2VP solutions are denoted as the A-set of solutions (Scheme 1). According to the recipe reported in the literature,^11^ precursor and perovskite solutions were prepared. 50 mg of PbBr_2_ was added in each of the PS-*b*-P2VP solutions. Stirring for varied periods of time was then performed on the PS-*b*-P2VP/PbBr_2_ solutions at room temperature (25 °C). The stirring rate was kept at 700 rpm. By the beginning of stirring, the added PbBr_2_ existed as precipitates in the solutions because toluene is a nonsolvent for PbBr_2_. However, after continuous and prolonged stirring, the solutions were turbid. The turbidity is due to Mie scattering, indicative of multiple emulsions occurring in toluene. This turbidity also indicates that PS-*b*-P2VP micelles could improve the dispersion of PbBr_2_ in toluene. Nevertheless, not all of PbBr_2_ was encapsulated by PS-*b*-P2VP micelles. Otherwise, the solutions should have appeared bluish rather than turbid. The mechanisms of multiple emulsions will be detailed in the following sections. At this stage, the milky solutions are denoted as the B-set of solutions (Scheme1). To remove nonencapsulated PbBr_2_, the B-set of solutions was centrifuged at 1000 rpm for 1 min. After centrifugation, the solutions appeared bluish. At the stage, the bluish solutions are denoted as a C-set of solutions (Scheme 1).

Because CsBr cannot be directly dissolved in toluene, CsBr was separately dissolved in methanol. A mass concentration of 15 mg/mL was prepared for CsBr in methanol. At this mass concentration, CsBr powders were well dissolved in methanol without precipitates. Thus, the CsBr solution using methanol is transparent. The transparent CsBr solutions are denoted as the D-set of solutions (Scheme 1). To produce perovskite nanocrystals, aliquots of the D-set of solutions were dripped into 1 mL of the C-set of solutions under stirring. The aliquots were 5, 10, 20, and 40 μL, respectively. The period of time of stirring was 5 h. Upon the addition of CsBr, the C-set of solutions becomes milky yellow. After 5 h of stirring, the milky yellow solutions were centrifuged at 8000 rpm (5 min) to remove perovskite microcrystals that cause Mie scattering and turbidity. The centrifuged solutions are denoted as the E-set of solutions (Scheme 1).

### Measurements and Analysis of Optical Properties

To understand structural evolution under multiple emulsions, we mixed PbBr_2_ with PS-*b*-P2VP micelles in the A-set of solutions for varied durations (0.5, 5, 10, 24, and 48 h). The solutions were then centrifuged at 1000 rpm for 1 min. The dynamic light scattering (DLS) measurements were performed using a ZEN2003 Zeta upgrade of MALVERN. UV–vis absorbance spectra were measured using a JASCO V-700 spectrophotometer. Photoluminescence quantum yield (PLQY) and photoluminescence (PL) spectra were measured utilizing a FluoroMax spectrometer (HORIBA Scientific). Operating at an excitation wavelength of 375 nm, the fluorescence was carefully observed. Time-resolved photoluminescence (TRPL) spectra were gathered to investigate the lifetime of triplet excitons. This was achieved using a photomultiplier (PMA-C-192-N-M, PicoQuant) and analyzed through a time-correlated single-photon counting board (TimeHarp 260). Employing a 375 nm picosecond pulsed laser diode (LDH-IB-375-B), the sample was excited and synchronized by a laser driver (Taiko PDL M1) with a repetition rate of 20 MHz, all operating under pulsed emission mode. Note that measurement of UV–vis absorption, PL, PLQY, and TRPL spectra needs diluted solutions. Thus, the solutions were diluted to one-thirtieth of their concentrations for measuring UV–vis absorption, PL, and PLQY spectra and to one-fortieth of their original concentrations for measuring TRPL spectra.

### Structural Characterization

Small-angle X-ray scattering (SAXS) and wide-angle X-ray scattering (WAXD) were performed at beamline TPS 13A of the Taiwan Photon Source at the National Synchrotron Radiation Research Center (NSRRC, Hsinchu). All of the solutions were sealed in 2 mm capillary tubes for the SAXS and WAXD measurements. The instrument configuration of TPS 13A has been detailed by Orion et al.^48^ According to the study of Orion et al.,^48^ we also performed background subtraction, calibration of intensity and sample-to-detector distance, and data reduction to obtain one-dimensional SAXS/WAXD profiles as a function of *q*. *q* is the scattering vector, defined by , where θ is the scattering angle. SAXS curves were further fitted by the SasView software.^49^

### Model Fitting

At a dilute concentration, micelles and nanoparticles in the solutions exhibit only form-factor scattering without structure-factor scattering. The form-factor scattering in the SAXS curves was fitted by the core–shell sphere model,^50−52^ unified exponential/power-law model (Beaucage model),^53,54^ or Porod model.^55^ The core–shell sphere model allows us to quantify the core radius, shell thickness, and scattering length densities (SLDs) of solvents, P2VP cores, and PS shells, respectively, described as follows:^50−52^
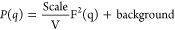
1
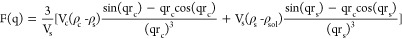
2

In (2), V_s_ denotes the volume of the whole sphere, V_c_ denotes the volume of the core, r_c_ denotes the radius of the core, r_s_ denotes the radius of the whole sphere (i.e., r_s_ = r_c_ + t_s_), and ρ denotes scattering length densities for solvent (superscript sol), core (superscript c), and shell (superscript s). For micelles with polydisperse size distribution, the function of Schultz particle distribution is additionally introduced in curve fitting to estimate the extent of polydispersity.

The Beaucage model allows us to quantify the radius gyration (*R*_g_) and power-law exponent for hierarchical structures, given by^53,54^
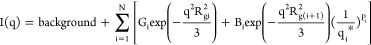
3with
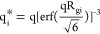
4

In (3) and (4), G_i_, B_i_, P_i_ and R_gi_ are Guinier prefactor, specific prefactor, power-law value, and Guinier radius of level-i aggregates.

The Porod model allows us to fit an intensity upturn with I ∼ q^–α^ at low *q*, given by^55^
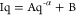
5

In (5), A and B are constants and α denotes the structural dimensionality.

### Morphological Observation and Elemental Analysis

Bright-field transmission electron microscopy (TEM) images were recorded using a JEOL JEM-F200 microscope performed at 200 kV, JEOL JEM-2100 performed at 120 kV, or Hitachi H-7500 performed at 80 kV. Selected area electron diffraction (SAED) patterns, energy-dispersive X-ray spectroscopy (EDX) 1D profiles, and 2D maps were also collected using a JEOL JEM-F200 microscope for quantitative analysis of topological structures, crystal lattices, and chemical elements. The solutions were dripped onto TEM copper grids placed on a filter paper each, which quickly adsorbed excess solvent. The TEM samples were dried at 25 °C for 24 h in a vacuum chamber.

## Results and Discussion

### Multiple Emulsions at the Stage of Mixing PbBr_2_ and PS-*B*-P2VP Micelles in Toluene

**Figure 1:**
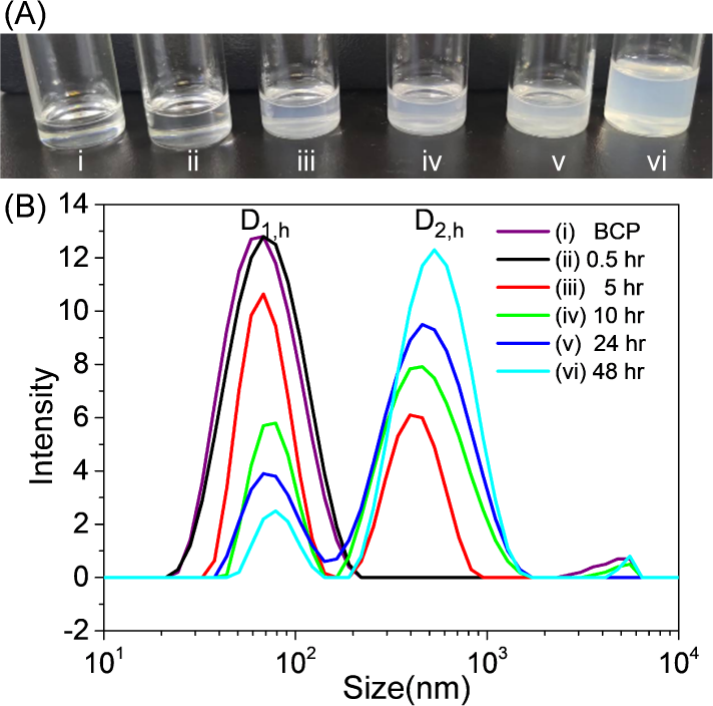
(A) Photos and (B) DLS data of A- and C-set solutions. The C-set solutions were obtained after centrifugation (1000 rpm, 1 min) of the B-set solutions, which were prepared by mixing PbBr_2_ (50 mg/mL) and PS-*b*-P2VP micelles (5 mg/mL) under stirring for various durations: (i) BCP, (ii) 0.5 h, (iii) 5 h, (iv) 10 h, (v) 24 h, and (vi) 48 h in toluene. “BCP” refers to a neat BCP solution without PbBr_2_.

Figure 1A demonstrates photos of neat BCP and its mixtures with PbBr_2_ powders dispersed in toluene. The photos were recorded for the A-set solution (Figure 1A_i_) and the C-set solutions (Figures 1A_ii_-1A_vi_), respectively. The solution containing only PS-*b*-P2VP in toluene was bluish, indicative of the formation of micelles (Figure 1A_i_).^45−47^ By addition and mixing PbBr_2_ powders with PS-*b*-P2VP micelles under stirring, the B-set of solutions appeared turbid before centrifugation (Figure S1A). In fact, toluene is a nonsolvent for PbBr_2_. Without PS-*b*-P2VP, PbBr_2_ always precipitates in toluene (Figure S1B). This comparison indicates that PS-*b*-P2VP micelles act as surfactants to improve the colloidal emulsions of PbBr_2_ in toluene, similar to hierarchical emulsions.^56^

Nevertheless, the turbidity of the B-set solutions indicates that PS-*b*-P2VP micelles induce colloidal emulsions of PbBr_2_ at the micro- and mesoscales (Figure S1A). The colloidal emulsions of PbBr_2_ with PS-*b*-P2VP micelles produce hierarchical structures. Of these hierarchical structures, microstructures lead to Mie scattering. Because the self-assembly of PS-*b*-P2VP micelles only produces mesoscale micelles, the formation of microscale structures should be due to the microemulsion of PbBr_2_ powders that are stabilized by PS-*b*-P2VP micelles.

To remove PbBr_2_ microcrystals, the B-set turbid solutions were further centrifuged at 1000 rpm for 1 min. Figure 1A_ii_–A_vi_ demonstrates that increasing the duration of mixing PbBr_2_ powders and PS-*b*-P2VP micelles tends to produce foggy solutions after centrifugation at 1000 rpm for 1 min. Particularly, the foggy extent increases with increasing stirring time.

Figure 1B demonstrates the DLS profiles collected for the C-set solutions containing PbBr_2_ and PS-*b*-P2VP micelles in toluene after centrifugation. The quantitative analysis of Figure 1B is summarized in Table 1. One noteworthy result is that neat PS-*b*-P2VP forms monodisperse micelles with a hydrodynamic diameter (*D*_1,h_) of approximately 61.4 nm. The mixtures of PS-*b*-P2VP and PbBr_2_ form micelles with a bimodal size distribution upon prolonged stirring (>0.5 h) in toluene, indicative of the formation of a secondary structure. Upon stirring for 5 h, a second size distribution (*D*_2,h_) appears at 400.8 nm. Furthermore, with increasing stirring time, both the DLS position and the intensity of *D*_2,h_ increase. After 48 h of stirring, *D*_2,h_ increases from 400.8 to 497.9 nm, indicating a size increase of 100 nm. In comparison, *D*_1,h_ slightly increases with increasing stirring time, while its DLS intensity significantly decreases over time. The changing trends of *D*_1,h_ and *D*_2,h_ seem to be correlated. As the PS-*b*-P2VP and PbBr_2_ mixtures are stirred in toluene, the secondary structures of *D*_2,h_ grow in size and proportion at the expense of the primary micelles of *D*_1,h_. The formation of the secondary structure is likely related to interactions between PbBr_2_ complexes and PS-*b*-P2VP micelles.

**Table 1:** A summary of *D*_1,h_ and *D*_2,h_ obtained from Figure 1B

materials/time (hr)	D_1,h_ (nm)	D_2,h_ (nm)
only PS-*b*-P2VP	61.4	0
PS-*b*-P2VP+PbBr_2_/0.5 h	64.5	0
PS-*b*-P2VP+PbBr_2_/5 h	66.5	400.8
PS-*b*-P2VP+PbBr_2_/10 h	73.7	427.7
PS-*b*-P2VP+PbBr_2_/24 h	76.5	441.3
PS-*b*-P2VP+PbBr_2_/48 h	77.8	497.9

**Figure 2:**
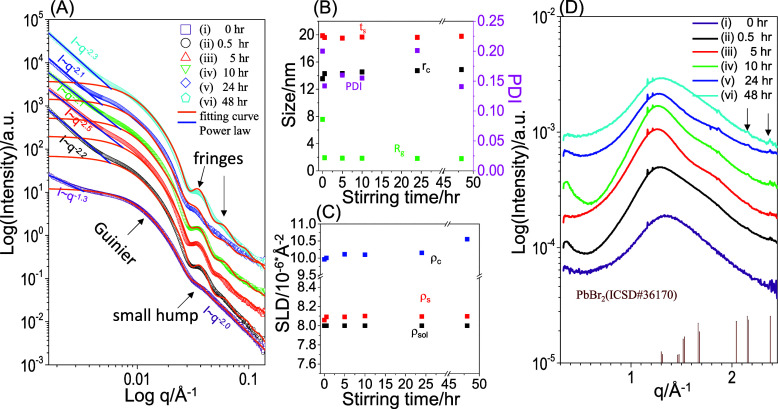
(A) SAXS (symbols: measured data; lines: fitted curves), (B) structural parameters, (C) SLD, and (D) WAXD data of A-set and C-set solutions. A standard powder diffraction pattern of PbBr_2_ orthorhombic lattices is shown in panel D for comparison. The A-set solution contains only neat PS-*b*-P2VP micelles. The C-set solutions contain mixtures of PbBr_2_ and PS-*b*-P2VP micelles, obtained after being stirred for various durations followed by centrifugation (1000 rpm, 1 min). Stick pattern in panel D: a standard of orthorhombic PbBr_2_ (ICSD#36170).

Several studies have demonstrated that simultaneous SAXS and WAXD characterization performed in reflection or transmission mode allows researchers to gain in-depth structural details of perovskite colloids or thin films at both the nanoscale and atomic scale.^57−61^ To understand structural details, we further characterized the BCP and precursor solutions by simultaneous SAXS and WAXD. Figure 2 shows SAXS and WAXD profiles measured for the hierarchical structures in solution along with structural parameters and SLD values. The structural parameters and SLD values were obtained by quantitatively analyzing the SAXS profiles (open symbols) through model fitting (solid lines). For neat PS-*b*-P2VP in toluene, the SAXS profile shows a low-*q* upturn with *I* ∼ *q*^–1^·^3^ in intensity at *q* < 0.003 Å^–1^, Guinier scattering in the *q* region of 0.003–0.03 Å^–1^, and a small hump at *q* > 0.03 Å^–1^ (Figure 2A). These scattering features correspond to core–shell micelles with a polydisperse size distribution, which can be well fitted by the model of spherical micelles with a core–shell structure. The dimensions fitted by the core–shell-sphere model are consistent with those values measured by DLS.

In comparison, the mixtures of PS-*b*-P2VP and PbBr_2_ in the centrifuged solutions show an intensity upturn at low *q* (<0.004 Å^–1^). The low-*q* upturn in intensity indicates the clustering of micelles. Due to the limited *q* resolution, the SAXS profiles only capture information on shape rather than size for the secondary structures of *D*_2,h_. Thus, the low-*q* upturn was fitted by the power-law model. The intensity of the low-*q* upturn decays with *I* ∼ *q*^–2^·^3^, indicating that the dimensionality of the secondary structures is intermediate between two- and three-dimensional. Furthermore, upon prolonged stirring, the small hump further develops into fringes. The well-defined fringes indicate that the micelles have a reduced size distribution after the addition of PbBr_2_, prolonged stirring, and centrifugation. Note that the high-*q* region (0.05–0.14 Å^–1^) shows an intensity decay with *I* ∼ *q*^–2^. This may arise from inhomogeneity within the swollen PS shells, which has been frequently observed for polymeric core–shell micelles.^51^

To quantify the micelles, the SAXS curves (Figure 2A) were fitted by using three models. The low-*q* (*q* < 0.004 Å^–1^) upturn in intensity corresponding to aggregates was fitted with the Porod model. The Guinier region and fringes were fitted using the core–shell polydisperse sphere model. Additionally, the high-*q* region was fitted with the Beaucage model to quantify the inhomogeneity. The orange lines shown in Figure 2A represent the fitted results based on the structural parameters [including *r*_c_ (core radius), *t*_s_ (shell thickness), *R*_g_ (inhomogeneity size), and PDI (polydispersity)], and ρ_c_, ρ_s_, and ρ_sol_ (SLD values of core, shell, and solvent), which are summarized in Figure 2B,C.

Figure 2B,C shows that upon continuous stirring, both *r*_c_ and ρ_c_ slightly increase with stirring time. In comparison, prolonged stirring time has no influence on *t*_s_, ρ_s_ or ρ_sol_. This comparison indicates that PbBr_2_ selectively interacts with P2VP cores during stirring. Note that the selective incorporation of PbBr_2_ into P2VP cores may stabilize the *R*_g_ of inhomogeneity. However, the stabilization mechanism remains unclear and is beyond the scope of this research. The PDI randomly fluctuates without showing any correlation with stirring time.

Figure 2D shows the corresponding WAXD profiles for the same solutions. The WAXD profiles display one intense halo along with two broad peaks of weak intensity in the high-*q* region (1.5–2.5 Å^–1^). The halo corresponds to inter- or intrachain correlations of PS-*b*-P2VP. Note that the two small but sharp peaks (at *q* = 1.165 and 1.215 Å^–1^) are not attributed to diffraction by PbBr_2_ orthorhombic lattices (ICSD#36170). These sharp peaks are likely due to the presence of trace impurities in the neat PS-*b*-P2VP micelles in toluene. Additional weak peaks at 2.148 and 2.379 Å^–1^ (indicated by arrows) are present, corresponding to PbBr_2_ orthorhombic lattices. The WAXD results indicate that PbBr_2_ microcrystals likely exist in a minor content within the solutions.

**Figure 3:**
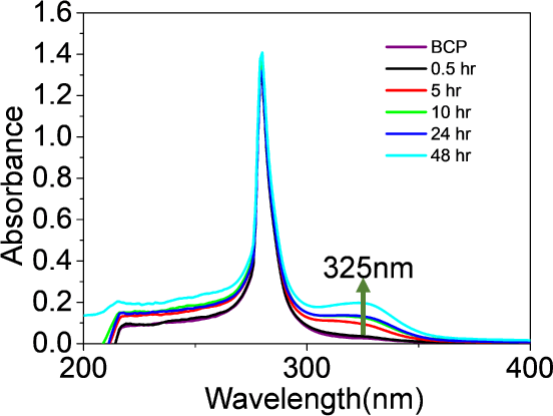
UV–vis absorbance spectra of A- and C-set solutions. The A-set solution contains only neat PS-*b*-P2VP micelles. The C-set solutions contain mixtures of PbBr_2_ and PS-*b*-P2VP micelles obtained after being stirred for various durations followed by centrifugation.

Figure 3 shows UV–vis absorbance spectra for the same solutions, diluted to one-thirtieth of their original concentrations. The spectra reveal an intense peak around 280 nm, attributed to toluene, and a shoulder band emerges near 325 nm for the mixtures of PS-*b*-P2VP and PbBr_2_ in toluene. The intensity of this shoulder band increases with longer stirring times.

The optical properties of PbBr_2_ and its complexes have been systematically studied by Kamat’s group,^10,62^ demonstrating that PbBr_2_ powders exhibit an absorbance band at 280 nm, solvated [PbBr_3_]^−^ complexes show an absorbance band at 310 nm, and [PbBr_3_]^−^ complexes bound with ligands absorb at 325 nm. According to these studies,^10,62^ the absorbance band at 325 nm can be assigned to [PbBr_3_]^−^ complexes interacting with P2VP chains. Note that the absorbance band of toluene (or PS-*b*-P2VP) is located at 280 nm, overlapping with the absorbance band assigned to PbBr_2_ powders.

The main absorbance peak at 280 nm and the shoulder at 325 nm behave differently. The absorbance shoulder at 325 nm shows a gradual increase in intensity with longer stirring times, while the main absorbance remains unchanged. This suggests that [PbBr_3_]^−^ complexes are present in significant amounts in solution, while PbBr_2_ microcrystals exist only in minor quantities. Furthermore, the [PbBr_3_]^−^ complexes are likely bound to P2VP chains. Supporting evidence from TEM and EDS is provided below.

**Figure 4:**
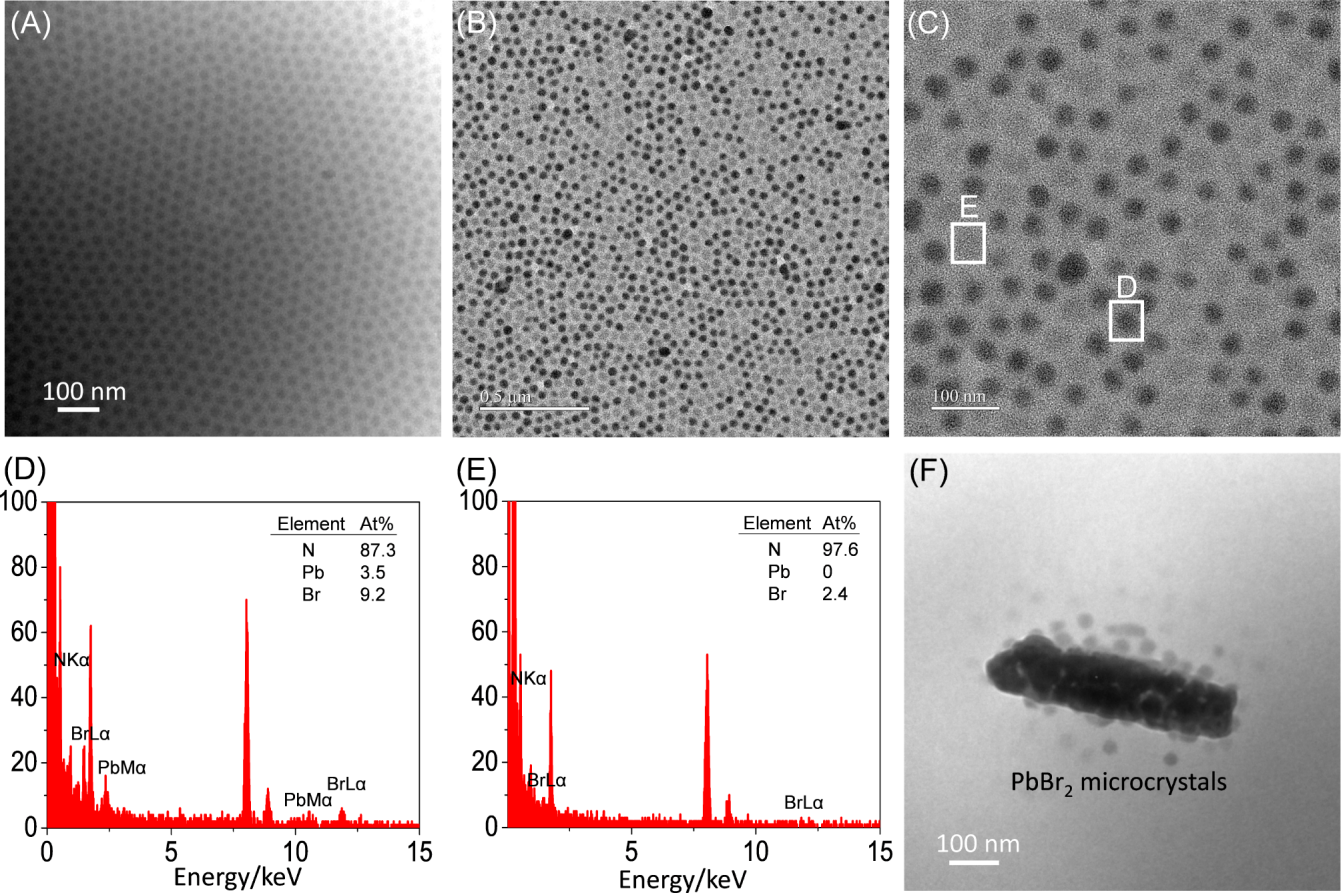
TEM images and EDS profiles acquired in the dried state for PS-*b*-P2VP micelles (A) without and (B–F) with the addition of PbBr_2_. Dried TEM samples were prepared after prolonged stirring (48 h) and subsequent centrifugation at 1000 rpm for 1 min. The solutions initially contained either neat PS-*b*-P2VP micelles or a mixture of them with PbBr_2_ in toluene.

UV–vis absorbance spectra reveal optical properties of solutions and not the morphologies of solutes in the solvent. To demonstrate the spatial distribution of PbBr_2_ microcrystals and [PbBr_3_]^−^ complexes, we further performed TEM and EDS characterization for some of the B-set solutions. TEM samples were prepared according to the method described in the experimental section. Figure 4 shows TEM images of neat PS-*b*-P2VP and its mixture with PbBr_2_ in a dried state. Two insights can be obtained by qualitatively analyzing the images.

First, neat PS-*b*-P2VP micelles ordered in dried state, where the P2VP cores appear with a higher contrast than the PS shells (Figure 4A). We further quantitatively analyzed the dimensions with ImageJ for the P2VP cores and intercore distance. Figure S2 demonstrates that the core diameter and intercore distance were approximately 29.9 ± 4.9 and 45 nm, respectively. A comparison between the core diameter and intercore distance indicates that the thickness of each PS shell was approximately 7.5 nm in dried state. In contrast, the PS shells are swollen by toluene and thus have an average thickness of 19.6 nm (Figure 2B).

Second, the mixture of PS-*b*-P2VP and PbBr_2_ also formed core–shell micelles (Figure 4B,C). The PS-*b*-P2VP micelles exhibit two colors with the addition of PbBr_2_: dark and gray. EDS 1D profiles of Pb, Br, and N demonstrate that the dark P2VP cores capture [PbBr_3_]^−^ complexes (Figure 4D), whereas the gray P2VP cores are enriched with only Br^–^ ions (Figure 4E). The corresponding high-magnification TEM image indicates that the [PbBr_3_]^−^ complexes encapsulated by P2VP cores are amorphous rather than crystalline (Figure S3). Furthermore, not all of the PS-*b*-P2VP micelles are occupied by abundant [PbBr_3_]^−^ complexes. Some of the PS-*b*-P2VP micelles are empty, with no capture of [PbBr_3_]^−^ complexes (Figure 4E). Occasionally, we observed a small amount of PbBr_2_ microcrystals with surfaces adsorbed by PS-*b*-P2VP micelles (Figures 4F and S4). This result suggests two prominent phenomena. First, most of the P2VP cores captured [PbBr_3_]^−^ complexes rather than PbBr_2_ microcrystals. Second, a small amount of PbBr_2_ fragments remained in the centrifuged solution, contributing to the diffraction peaks at *q* = 2.148 and 2.379 Å^–1^ observed in Figure 2D. These peaks, corresponding to the orthorhombic lattices of PbBr_2_, become more noticeable in solutions stirred for extended periods of time. A possible explanation is that stirring promotes PbBr_2_ complexation, leading to the formation of [PbBr_3_]^−^ complexes at the interface between PS-*b*-P2VP micelles and PbBr_2_ microparticles. As a result, the PbBr_2_ microparticles act as a “reservoir” for the formation of these complexes, and the complexation gradually reduces the size of some PbBr_2_ microparticles. Figures 2D and 4F indicate that these PbBr_2_ fragments are not entirely removed by the first centrifugation.

Furthermore, some micelles were observed to cluster in addition to the presence of individual micelles, as shown in Figure S5. When clustering occurs, the micelles adopt deformed shapes and exhibit size variations. These findings are displayed in Figures 4, S4, and S5, which are consistent with the distribution of individual micelles and clustered micelles, as discussed in the quantitative analysis of the DLS spectra (Figure 1B).

### Contents of Methanol and CsBr on the Nucleation and Growth and PL Performance of Perovskite Crystals

**Figure 5:**
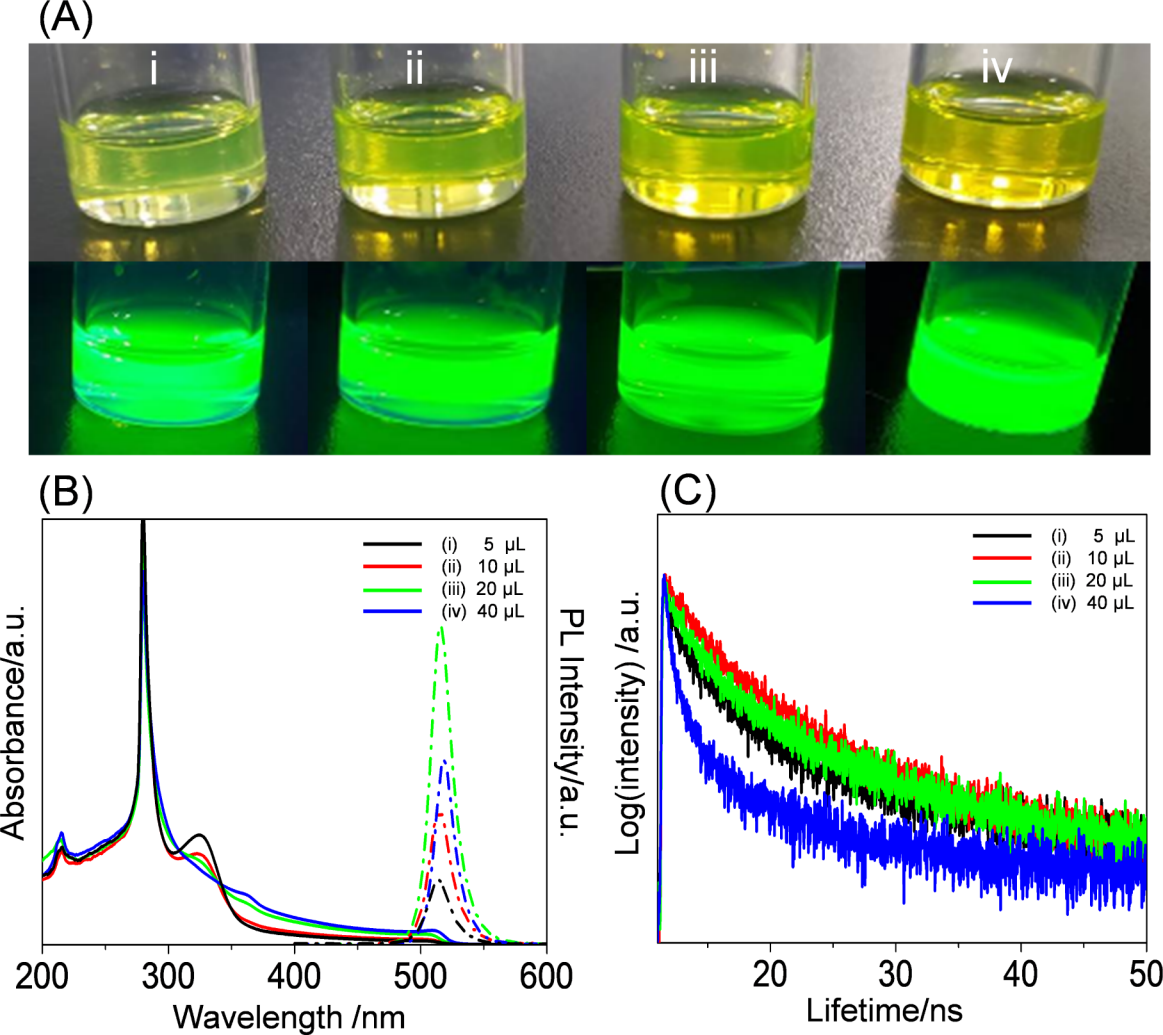
(A) Photos, (B) UV–vis absorbance/PL spectra, and (C) TRPL spectra of E-set solutions that were prepared by adding different amounts [(i) 5, (ii) 10, (iii) 20, and (iv) 40 μL] of a D-set solution (15 mg/mL CsBr in methanol) into a C-set solution that initially contained 50 mg/mL PbBr_2_ and 5 mg/mL PS-*b*-P2VP under prolonged stirring (48 h) in 1 mL toluene before centrifugation (1000 rpm, 1 min). Postcentrifugation (8000 rpm, 5 min) was performed on the E-set solutions before measuring their optical properties.

Because CsBr has low solubility in toluene, we separately dissolved CsBr in methanol to prepare a D solution with a mass concentration of 15 mg/mL. To prepare CsPbBr_3_ nanocrystals in E-set solutions, 5, 10, 20, or 40 μL of D-set solution (15 mg/mL CsBr in methanol) were respectively added to C-set solutions that were obtained after 48 h stirring of PbBr_2_ and PS-*b*-P2VP in toluene and then centrifuging at 1000 rpm (1 min). Centrifugation was further imposed on the E-set solutions to remove unencapsulated CsPbBr_3_ microcrystals. After postcentrifugation, the E-set solutions appeared fluorescent green and emitted PL under λ_excite_ = 365 nm. Figure 5B shows UV–vis absorbance spectra (in the range 300–600 nm) of the E-set solutions that had been centrifuged at 8000 rpm (5 min). Figure 5B demonstrates three absorbances centered at 325, 360, and 515 nm, respectively. The three absorbance bands are assigned to [PbBr_3_]^−^ complexes, [PbBr_4_]^2–^ complexes, and CsPbBr_3_ nanocrystals.^62^ The absorbance intensity of [PbBr_3_]^−^ complexes decreases with the increase in the content of D-set solution, but the absorbance intensities of [PbBr_4_]^2–^ complexes and CsPbBr_3_ nanocrystals increase. The trend displayed in Figure 5B indicates that adding more content of D-set solution produced more [PbBr_4_]^2–^ complexes and CsPbBr_3_ nanocrystals, growing at the expense of the [PbBr_3_]^−^ complexes. Furthermore, the PL intensity is correlated with the intensity of the absorbance centered at 515 nm. This correlation indicates that the PL intensity is mainly determined by the quantity of CsPbBr_3_ nanocrystals.

Nevertheless, the CsPbBr_3_ nanocrystals that were obtained by adding 10 μL of D solution display higher PLQY and longer decay time than those nanocrystals that were obtained by adding 5, 20, or 40 μL of D solution initially having a mass concentration of 15 mg/mL for CsBr in methanol (see Table 2). This result indicates that although adding more D solution produced more CsPbBr_3_ nanocrystals, the quality of the CsPbBr_3_ nanocrystals was not the best. Particularly, the CsPbBr_3_ nanocrystals obtained by adding 40 μL of D solution have the worst PLQY and the shortest decay time. Moreover, these nanocrystals formed by adding 40 μL of D solution decayed quickly after 4 weeks of storage (Figure S6) at ambient conditions without controlled humidity. In contrast, the CsPbBr_3_ crystals obtained by adding 10 μL of D solution still exhibit PL after 4-week storage.

**Table 2:** Optical Properties of the E-set Solutions are Summarized from Figure 5A^a^

CsBr (in methanol)	Added aliquots of a D-solution	PLQY %	Wavelength nm	Average lifetime (ns)
15 mg/mL	5 μL	80.99	516	11.79
15 mg/mL	10 μL	84.46	516	14.32
15 mg/mL	20 μL	73.40	516	12.9
15 mg/mL	40 μL	29.09	520	5.88

a PLQY spectra in the range of 370-600 nm were measured at a speed of 300 nm/min under an excitation wavelength of 375 nm

To further investigate the effects of methanol content, we prepared an additional series of D-set solutions with varying CsBr concentrations in methanol: 15, 7.5, and 3.75 mg/mL. Perovskite supernatants were prepared by adding different aliquots of these solutions to the precursor supernatants, ensuring a consistent CsBr content: 10 μL for 15 mg/mL, 20 μL for 7.5 mg/mL, and 40 μL for 3.75 mg/mL. While the total CsBr content remained constant, the methanol content varied, allowing us to isolate the effect of methanol on micelle stability independent of CsBr concentration. The perovskite supernatants were characterized by using UV–vis absorbance, PL, PLQY, and TRPL spectroscopy, and their long-term stability was also tested. As shown in Figure S7 and Table S1, adding 40 μL of methanol with 3.75 mg/mL CsBr produced a perovskite solution with high PL but low PLQY, shorter lifetime, and poor long-term PL stability. In contrast, adding a 10 μL aliquot of 15 mg/mL CsBr in methanol resulted in a solution with lower PL but higher PLQY, longer lifetime, and superior long-term PL stability. This suggests that a higher methanol content increases the quantity of CsPbBr_3_ nanoparticles but reduces the overall quality.

**Figure 6:**
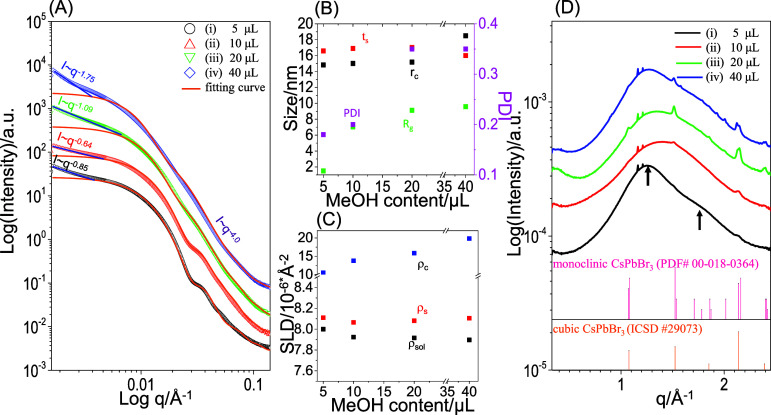
(A) SAXS profiles (symbols: measured data; lines: fitted curves), (B) structural parameters, (C) SLD values, and (D) WAXD profiles of the fresh E-set solutions that were prepared by adding different contents [(i) 5, (ii) 10, (iii) 20 and (iv) 40 μL] of a D-set solution (15 mg/mL CsBr in methanol) in centrifuged C-set solutions. 48-h stirring and centrifugation were performed on the C-set solutions containing mixtures of PbBr_2_ and PS-*b*-P2VP before the addition of CsBr. Stick patterns in (D): standard profiles of cubic CsPbBr_3_ (ICSD#29073) and monoclinic CsPbBr_3_ (PDF# 00–018–0364).

To understand the structural evolution of PS-*b*-P2VP micelles and CsPbBr_3_ nanocrystals, we further characterized the E-set solutions by using SAXS and WAXD. Figure 6 shows SAXS and WAXD spectra for the freshly prepared E-set solutions without storage. Two scattering features are present in the SAXS spectra (Figure 6A). The first feature displays a combination of a small intensity upturn at *q* < 0.003 Å^–1^, Guinier-like scattering in the region of q = 0.003–0.02 Å^–1^, and scattering fringes at *q* > 0.02 Å^–1^ (Figure 6A_i_–6A_iii_). This scattering feature is similar to that described in Figure 2. This result indicates that PS-*b*-P2VP micelles still maintain a monodisperse size distribution and a core–shell spherical shape in the E-set solutions, which were prepared by adding 5–20 μL of a D-set solution to C-set solutions, respectively.

In comparison, adding a higher content of the D-set solution into C-set solutions produced E-set solutions that display SAXS profiles with dampened fringes and a strong low-*q* upturn (Figure 6A_iv_). It has been well demonstrated that scattering fringes are related to size distribution^50−52^ and that a low-*q* upturn indicates structural clustering.^55^ Therefore, the dampening of the scattering fringes and the strong low-*q* upturn suggest that the PS-*b*-P2VP micelles become polydisperse and largely aggregate when a high content of 40 μL of a D-set solution is added to the C-set solution. Note that the high-*q* region shows an intensity decay with *I* ∼ *q*^–4^, indicating that the high-*q* region is mainly scattered by perovskite nanoparticles.

To quantify the micelles and perovskite nanoparticles, the SAXS curves shown in Figure 6A were further fitted by the model. The Guinier region and fringes were fitted by the model of polydisperse core–shell spheres to obtain the structural details for micelles. The high-*q* regions were fitted by the Beaucage model to obtain the dimensionality and size of CsPbBr_3_ nanoparticles. The lines shown in Figure 6A represent the fitted results based on the structural parameters summarized in Figure 6B,C. Figure 6B,C demonstrates that adding more CsBr into the centrifuged C-set solutions gradually increases the SLD (ρ_c_) and size (*r*_c_) of cores. Furthermore, the size (*R*_g_) of the CsPbBr_3_ nanoparticles can be increased by adding more CsBr. The increased SLD and size of cores correlated with the increased size of CsPbBr_3_ nanoparticles, indicating that CsPbBr_3_ nanoparticles indeed selectively grow inside the P2VP cores. Nevertheless, the growth of large CsPbBr_3_ nanoparticles may deform or distort the micelles, resulting in an increase in the size dispersity of micelles. Note that the P2VP cores only increase by 3 nm in diameter at most, while the CsPbBr_3_ nanoparticles largely increase from *R*_g_ = 1.5 nm (upon adding 10 μL of a CsBr solution using methanol) to *R*_g_ = 9.6 nm (upon adding 40 μL of a CsBr solution using methanol). This discrepancy suggests that the growth of large CsPbBr_3_ nanoparticles may extend over several micelles. Furthermore, the deformation and distortion of micelles are responsible for an increase in the PDI.

Figure 6D shows the corresponding WAXD profiles for the E-set solutions, featuring two broad halos indicated by thick vertical arrows. These broad halos are related to the inter- and intrachain correlations of PS-*b*-P2VP chains or intercluster correlations of complexes. Such broad halos are frequently observed in hybrids of polymers and inorganic nanomaterials.

In addition, two small diffractions with weak intensity are present, centered at specific *q*-values (1.5117 and 2.1350 Å^–1^) for the perovskite solutions that were prepared by adding small contents (<20 mL) of CsBr in centrifuged precursor solutions. These small diffractions can be assigned to the signals of CsPbBr_3_ cubic lattices (ICSD#29073). In comparison, adding 40 μL of a CsBr solution produced monoclinic lattices (PDF#00-018-0364). This comparison indicates that CsPbBr_3_ monoclinic lattices predominantly grew in the E-set solution to which 40 μL of a D-set solution was added whereas CsPbBr_3_ cubic lattices predominantly grew in the E-set solutions to which 5–20 μL of a D-set solution was added.

**Figure 7:**
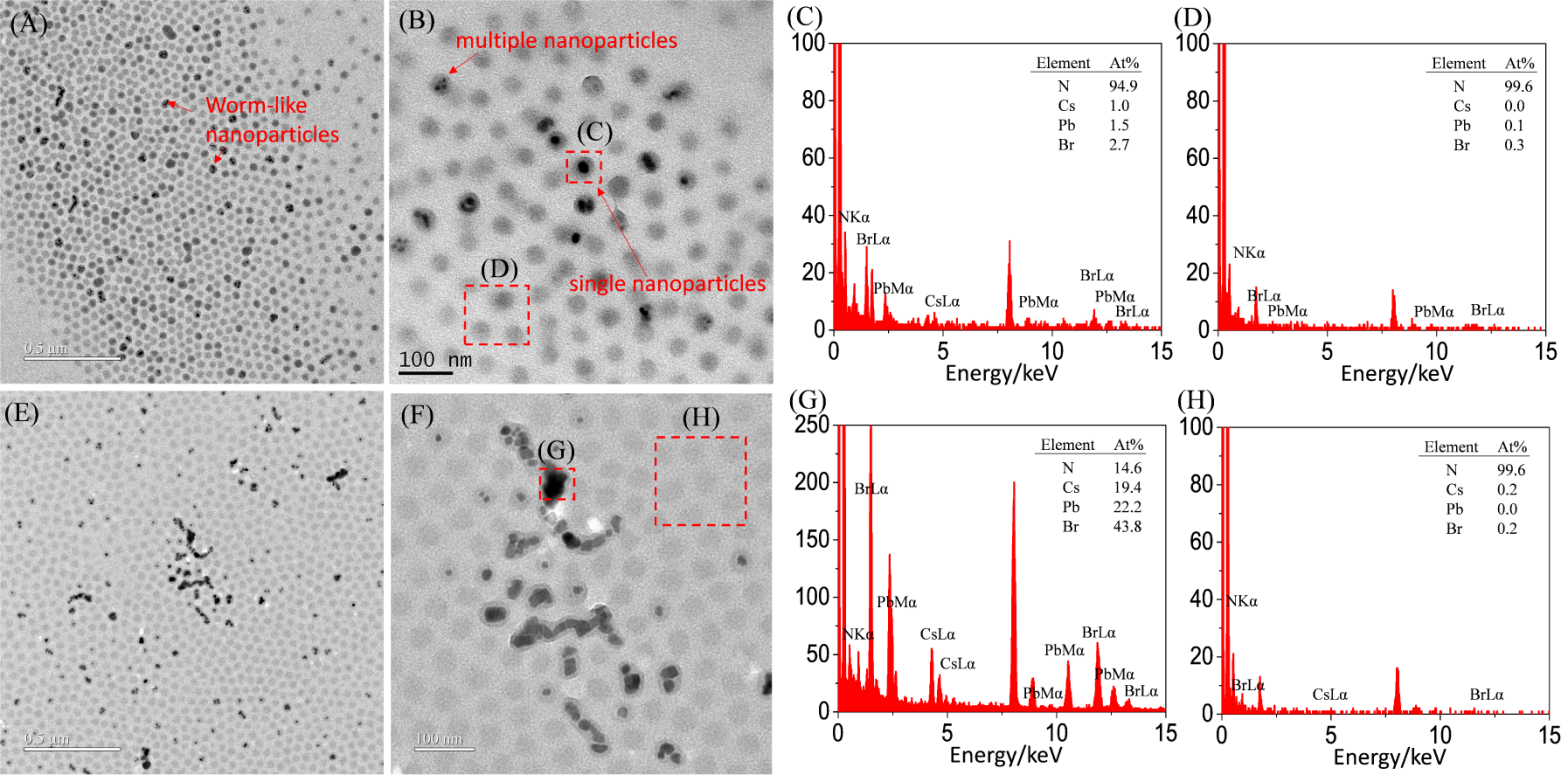
(A, B, E, F) Low- and high-magnification TEM images and (C, D, G, H) EDS profiles. The data were measured on a dried state of two fresh E-set solutions with addition of (A–D) 10 and (E–H) 40 μL of a D-set solution into C-set solutions. The positions selected for EDS characterization are marked by dashed boxes in panels B and F, respectively.

To probe structural details, we further performed TEM and EDS characterizations on the E-set solutions. Figure 7 shows representative TEM images and EDS spectra measured on a dried state of the E-set solutions that are previously discussed for Figure 6A_ii_/D_ii_,6A_iv_/D_iv_. Figure 7A,B shows a morphology of dark and gray micelles. The dark micelles are due to tiny nanoparticles growing inside the PS-*b*-P2VP micelles. The tiny nanoparticles were encapsulated by individual micelles. Furthermore, multiple nanoparticles or single nanoparticles grew inside individual micelles (Figure 7B). Note that a few CsPbBr_3_ nanocrystals grew cross several micelles to form worm-like nanoparticles (indicated by red arrows in Figure 7A). The formation of worm-like nanoparticles is accompanied with the deformation and fusion of the P2VP cores in PS-b-P2VP micelles that grew the worm-like nanoparticles.

The EDS profile of a selected tiny single nanoparticle indicates that its composition consists of Cs, Pb, and Br with a ratio of 1.0:1.5:2.7 (Figure 7C). This ratio suggests that the tiny nanoparticle should be a CsPbBr_3_ nanocrystal. In comparison, the EDS profile (Figure 7D) of the selected gray micelles shows strong N signals but weak Br signals and even weaker Pb signals, with no detectable Cs^+^ ions. This result suggests that the gray micelles are primarily composed of P2VP chains, with only a small but distinguishable amount of Pb and Br elements present.

Figure 7A–D indicates that adding 10 μL of a D-set (CsBr in methanol) solution into a C-set solution formed a low number density of tiny CsPbBr_3_ nanoparticles. The tiny CsPbBr_3_ nanoparticles grew at the expense of [PbBr_3_]^−^ complexes encapsulated by individual micelles and Cs^+^ ions that diffused from a liquid medium to P2VP cores through the PS shell. In addition, the crystal growth is not uniform, leading to multiple nanoparticles or single nanoparticles simultaneously growing inside individual P2VP cores. Furthermore, because of an unbalanced molar ratio of PbBr_2_ to CsBr in the E-solution, not all of the [PbBr_3_]^−^ complexes could form CsPbBr_3_ crystals through interactions with a low content of Cs^+^ cations. The scarcity of Cs^+^ cations with respect to the content of the [PbBr_3_]^−^ complexes suggests that there should be Pb and Br residues within P2VP cores. The Pb and Br residues can be detected by EDS (Figure 7D).

In comparison, CsPbBr_3_ crystals grew differently in the E-set solution with the addition of 40 μL of a D-set solution in a C-set solution. Figure 7E,F demonstrates that several worm-like nanoparticles grew cross multiple micelles. The EDS profile of a selected worm-like nanoparticle indicates an atomic ratio of Cs:Pb:Br = 19.4:22.2:43.8 (Figure 7G). This ratio suggests that the large nanoparticle should comprise CsPbBr_3_ crystals with abundant defects. This growth is different from the isolated growth of multiple CsPbBr_3_ nanoparticles or single CsPbBr_3_ nanoparticles inside of individual micelles. Furthermore, the gray micelles show no traces of [PbBr_3_]^−^ complexes (Figure 7H). This result indicates that excess [PbBr_3_]^−^ complexes cannot be retained in individual micelles in the E-set solution with the addition of 40 μL of a D-set solution. In other words, excess [PbBr_3_]^−^ complexes can “escape” from the capture of individual micelles. We believe that the formation of the large nanoparticles that cross multiple micelles should also involve excess [PbBr_3_]^−^ complexes that escaped from the capture of individual micelles. The formation of large nanoparticles crossing several micelles and the escape of [PbBr_3_]^−^ complexes from individual micelles should be linked with a decrease in micellization strength as the result of a high content of methanol in the E-set solution. The trends observed for the 10 and 40 μL cases also apply to the 5 and 20 μL samples (Figure S8). A comparison between Figures 7 and S8 shows that a 40 μL aliquot of CsBr/methanol resulted in the formation of fused nanoparticles, while 5, 10, and 20 μL aliquots led to the formation of individual encapsulated nanoparticles. Note that the sample prepared with the 20 μL aliquot contained a mixture of predominantly individual encapsulated nanoparticles with a minor content of fused nanoparticles (Figure S8D).

**Figure 8:**
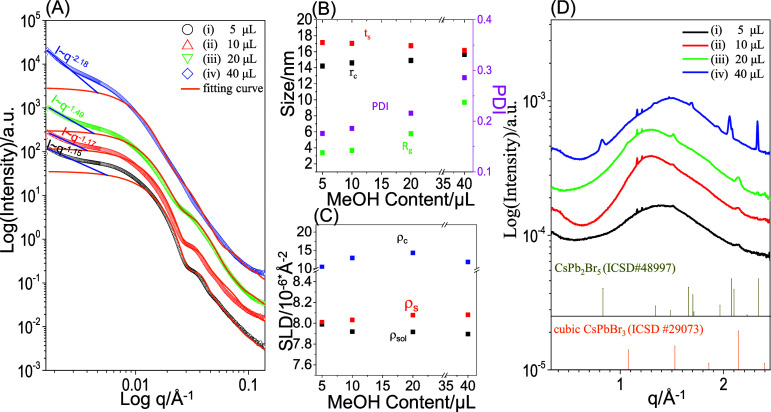
(A) SAXS profiles (symbols: measured data; lines: fitted curves), (B) structural parameters, (C) SLD values, and (D) WAXD profiles of the E-set solutions prepared by adding different volumes [(i) 5, (ii) 10, (iii) 20, and (iv) 40 μL] of a D-set solution (15 mg/mL CsBr in methanol) to centrifuged C-set solutions. The C-set solutions were prepared by stirring PbBr_2_ (50 mg/mL) and PS-*b*-P2VP (5 mg/mL) for 48 h in toluene and then centrifuged at 8000 rpm for 5 min. SAXS and WAXD characterizations were performed on supernatants after a four-week storage period of the E-set solutions. Stick patterns in panel D: standard profiles of cubic CsPbBr_3_ (ICSD#29073) and tetragonal CsPb_2_Br_5_ (ICSD#48997).

To trace the structural evolution of micelles and crystals to understand structural stability, we further characterized the E-set solutions with SAXS and WAXD after 4 weeks of storage. A comparison between Figures 6A and 8A indicates that prolonged storage has no significant impact on the scattering features associated with PS-*b*-P2VP micellization. Figure 8B,C shows structural parameters used for curve fitting to obtain the lines shown in Figure 8A. A comparison between Figure 8B,C and Figure 6B,C shows that for the E-set solution with the addition of 40 μL of CsBr solution using methanol, the SLD of the P2VP cores decreases from 19.8 × 10^–6^ to 11.8 × 10^–6^ Å^–2^. This decrease indicates that some crystals escape from P2VP cores after the four-week storage.

Figure 8D shows the corresponding WAXD profiles for the E-set solutions after storage for 4 weeks. Figure 8D shows that some of the E-set solutions exhibit different diffraction patterns upon prolonged storage, which cannot be assigned to CsPbBr_3_ monoclinic lattices (see Figure 8D_iv_). Several additional diffraction peaks newly emerge in the WAXD profile of the E-set solution containing the highest content of methanol (Figure 8D_iv_). These new diffractions can be assigned to the diffraction peaks of CsPb_2_Br_5_ tetragonal lattices (ICSD#48997). This result indicates that CsPbBr_3_ cubic lattices can be stable with the addition of 10 μL of D-set solution, but CsPbBr_3_ monoclinic lattices cannot remain stable when the amount of D-set solution added is higher or lower than 10 μL. The instability of CsPbBr_3_ monoclinic lattices eventually produces white precipitates of CsPb_2_Br_5_ tetragonal lattices (Figure S9). The CsPb_2_Br_5_ tetragonal lattices grew at an expense of [PbBr_3_]^−^/[PbBr_4_]^2–^ complexes and CsPbBr_3_ monoclinic lattices. This causes decreases in intensity for the absorbance bands assigned to [PbBr_3_]^−^/[PbBr_4_]^2–^ complexes and CsPbBr_3_ monoclinic lattices in the centrifuged E-set solutions that initially contained a high aliquot of methanol (Figure S10).

**Figure 9:**
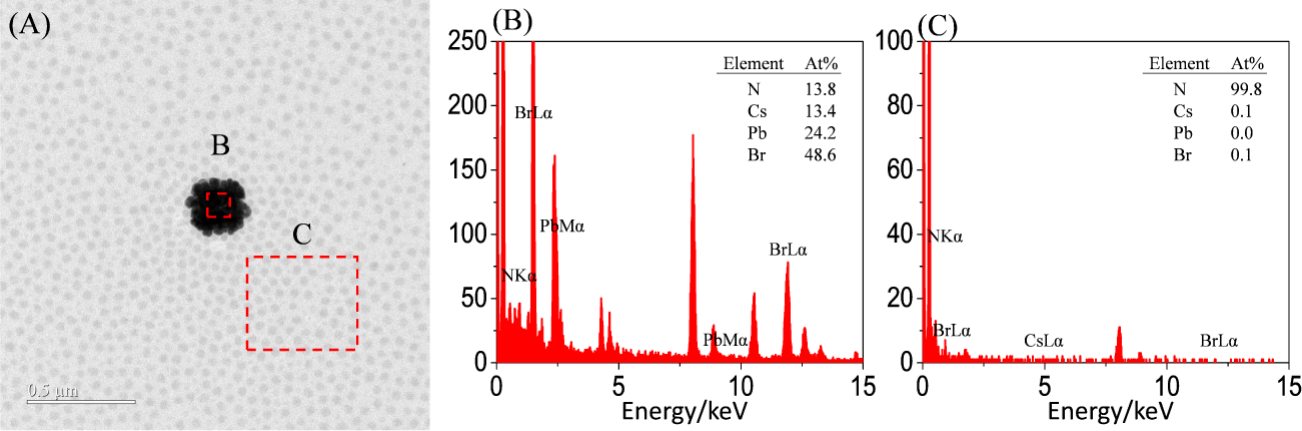
(A) TEM image and (B, C) EDS profiles measured in a dried stage of a precursor solution added with a 40 μL aliquot of CsBr solution after four-week storage.

We further characterized the dried state of dispersed crystals in the E-set solution containing 40 μL of the D-set solution by TEM and EDS. Figure 9A shows the coexistence of a large particle and PS-*b*-P2VP micelles. The large crystal exhibits a square shape, which results from the coarsening of irregular crystal aggregates. Quantitative analysis of the corresponding EDS spectrum of the large particle indicates that it exhibits an atomic ratio of Cs:Pb:Br of 13.4:24.2:48.6, similar to the composition of CsPb_2_Br_5_ crystals (Figure 9B). The EDS result (Figure 9B) aligns with the WAXD result (Figure 8D_iv_), confirming that the instability of CsPbBr_3_ monoclinic lattices produced CsPb_2_Br_5_ tetragonal lattices. Furthermore, the surface of the large crystal is enriched with PS-*b*-P2VP micelles. The adsorption of micelles onto the crystal explains why the EDS spectrum of the large crystal exhibits an atomic percentage of N signals of ∼13.8%. This result also indicates that the CsPb_2_Br_5_ crystal is so large that it cannot be solely encapsulated by a PS-*b*-P2VP micelle.

Although the dispersion of CsPb_2_Br_5_ crystals can be stabilized by the surface adsorption of a collection of micelles, the dimensional growth of CsPb_2_Br_5_ crystals cannot be prevented by surface adsorption of PS-*b*-P2VP micelles. As a result, CsPb_2_Br_5_ crystals could further grow during the prolonged storage. Once the dimensions of CsPb_2_Br_5_ crystals exceed a critical value where Brownian motion cannot preserve them, supernatants start to precipitate. Note that none of the micelles contained [PbBr_3_]^−^ complexes (Figure 9C). This result indicates that the consumption of [PbBr_3_]^−^ complexes likely occurs when CsPbBr_3_ monoclinic lattices or CsPb_2_Br_5_ tetragonal lattices form.

**Figure 10:**
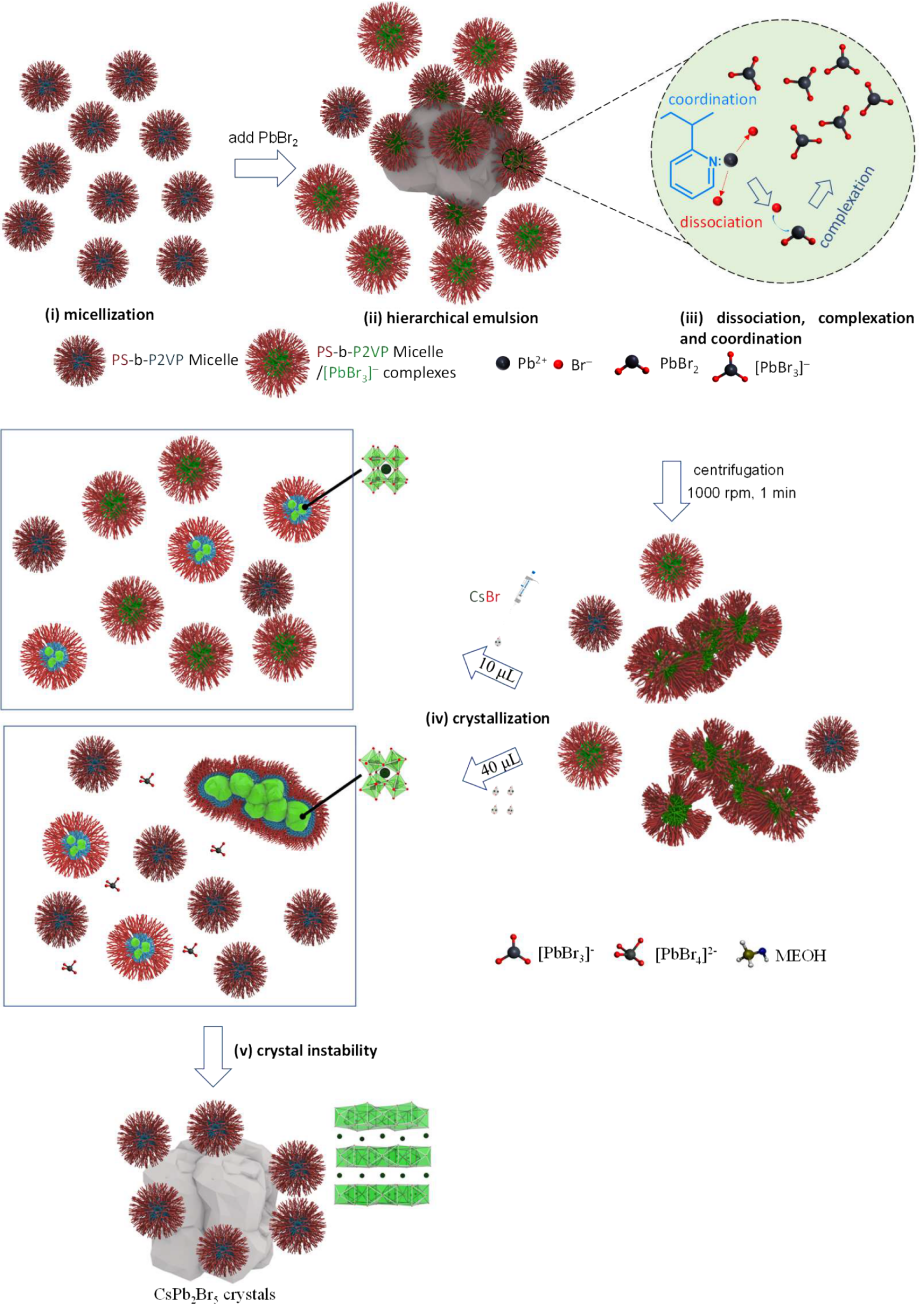
Schematic illustration of (i) PS-*b*-P2VP micellization, (ii) hierarchical emulsion, (iii) PbBr_2_ complexation and coordination interaction, (iv) crystallization of crystals, and (v) crystal instability proposed for CsPbBr_3_ nanocrystals templated via PS-*b*-P2VP colloids.

We have demonstrated the impacts of toluene, methanol, and PS-*b*-P2VP on the formation of ionic complexes and nanocrystals of cesium lead bromide. Figure 10 illustrates (i) PS-*b*-P2VP micellization; (ii) hierarchical emulsion; (iii) PbBr_2_ dissociation, complexation, and coordination interaction between P2VP and complexes; (iv) crystallization; and (v) crystal instability. Toluene (solubility parameter, δ = 18.2 MPa^1/2^)^63^ is a good solvent for PS (δ = 18.6 MPa^1/2^)^63^ but a nonsolvent for P2VP (δ = 21.3 MPa^1/2^),^64^ lead bromide, and cesium bromide. In neat toluene, PS-*b*-P2VP formed spherical micelles, all of which have P2VP cores and PS shells (Figure 10).

When PbBr_2_ was added in solutions containing PS-*b*-P2VP spherical micelles in toluene, multiple emulsion took place to produce turbid solutions (Figure 10_ii_). By multiple emulsions (a combination of microemulsion and nanoemulsion), PbBr_2_ became well dispersed in toluene. The microemulsion is analogous to Pickering emulsion.^56,65^ PS-*b*-P2VP micelles are adsorbed onto the surface of PbBr_2_ powders so that large PbBr_2_ powders can be well dispersed in toluene.

Nanoemulsion occurred through a series of dissociation, complexation, and coordination (Figure 10_iii_). To reduce incompatible contact with toluene, lead bromine formed ionic complexes with pyridine groups inside the P2VP cores through coordination interactions. The coordination interactions require PbBr_2_ dissociation, by which some of added PbBr_2_ broke down into its constituent, lead ions (Pb^2+^) and bromide ions (Br^–^). The PbBr_2_ dissolution was followed by coordination interactions of lead ions with lone pairs of electrons in P2VP. The coordination interactions are accompanied by the presence of excess bromide ions released from partial PbBr_2_ dissolution in solutions. The excess bromide ions further combine with undissolved PbBr_2_ to form [PbBr_3_]^−^ ionic complexes. Because toluene is a nonsolvent and [PbBr_3_]^−^ ionic complexes are small compared to PbBr_2_ powders, [PbBr_3_]^−^ ionic complexes can be easily encapsulated by PS-*b*-P2VP micelles. Furthermore, increasing the duration of stirring can increase the extent of the nanoemulsion. In contrast, dissociation, complexation, and coordination cannot occur if neat PbBr_2_ or its mixtures with a homopolymer (PS or P2VP) were added in toluene (Figure S11). The reason is that dissociation, complexation, and coordination need the good dispersion of PbBr_2_ in toluene through multiple emulsion where the PS shells impart steric repulsion for colloidal dispersion of PbBr_2_ in toluene. Note that the complexation and dissociation of PbBr_2_ induced by PS-*b*-P2VP occur at a slow rate at room temperature in toluene. Prolonged stirring at room temperature is necessary to increase the content of the [PbBr_3_]^−^ complexes captured by PS-*b*-P2VP micelles. Although temperatures and stirring rates critically influence PbBr_2_ complexation (Figures S12 and 13), our control experiments demonstrate that stirring at 700 rpm at 25 °C for hierarchical emulsion and PbBr_2_ complexation allows us to synthesize a good quality of CsPbBr_3_ nanoparticles (cf. Figures 5 and S14).

Centrifugation is needed to separate pristine PbBr_2_ microcrystals after prolonged stirring is performed to mix PbBr_2_ and PS-*b*-P2VP in toluene (Figure 10_iii_). The first-stage centrifugation is to prevent the formation of perovskite crystals directly from PbBr_2_ microcrystals that are stabilized by the adsorption of PS-*b*-P2VP. After the first-stage centrifugation to remove PbBr_2_ microcrystals, only encapsulated [PbBr_3_]^−^ complexes remain in toluene. Nevertheless, we found that not all of the PS-*b*-P2VP micelles in toluene capture [PbBr_3_]^−^ complexes. [PbBr_3_]^−^-free micelles also exist in toluene.

Furthermore, the selective incorporation of [PbBr_3_]^−^ into P2VP cores can increase the incompatibility between PS shells and complex-bound P2VP cores. Ye et al. demonstrated^66^ that increasing the incompatibility between PS and P2VP by tuning the quality of a cosolvent favors the formation of patchy micelles. Unlike conventional core–shell micelles, which have a more uniform layer of corona chains, patchy micelles have a nonuniform layer of corona chains. The patchy layer of corona chains is to avoid unfavorable contacts with core chains. As a result, such patchy micelles have anisotropic interactions for intermicelle clustering. The intermicelle clustering is energetically favorable.

CsBr cannot dissociate to form ions in toluene because of its solubility limitation in toluene. It is unlikely to form encapsulated CsPbBr_3_ nanoparticles by directly mixing CsBr and PbBr_2_ in toluene (Figure S15). Thus, CsBr must be separately dissolved in methanol in advance before being mixed with micelle-encapsulated complexes or micelle-adsorbed PbBr_2_ powders. The main reason is that CsBr has a good solubility in methanol as compared with dimethylformamide (DMF) and acetic acid (AC) (Figure S16). The good solubility of CsBr in methanol is critical to produce CsPbBr_3_ nanoparticles with superior PL. Using DMF, AC and a methanol/AC mixture to prepare CsBr solutions produce E-set solutions with low PL properties. (Figures S17 and 18). Note that methanol has a neutral pH, similar to that of deionized water (Figure S19). There are two benefits gained from using neutral methanol. Neutral methanol can effectively solvate Cs^+^ cations and Br^–^ anions. Furthermore, methanol is miscible with toluene. Therefore, methanol-solvated Cs^+^ cations can quickly bind with [PbBr_3_]^−^ complexes, thus accelerating the nucleation and growth of cubic or monoclinic crystals in a toluene/methanol cosolvent (right-top panel in Figure 10_iv_).

However, the content of methanol should be carefully controlled. Although using methanol favors binding of Cs^+^ cations with [PbBr_3_]^−^ complexes and quick crystal formation, using a high content of methanol weakens the micellization of PS-*b*-P2VP (Figure 10_ii_). Methanol (δ = 29.7 MPa^1/2^)^63^ is a good solvent for P2VP but a nonsolvent for PS. Upon adding a high content of methanol, the toluene/methanol cosolvent becomes a neutral solvent, thus reducing the segregation strength of the PS and P2VP blocks (right-bottom panel in Figure 10_iv_). The weakened segregation strength results in loose PS shells with a weak repulsion so that micelles likely fuse or aggregate together. Fused micelles or aggregated micelles offer a transportation path for anisotropic coalescence of CsPbBr_3_ monoclinic crystals. This reason explains why anisotropic, irregular nanocrystals grew across several micelles. It has been demonstrated that the growth of anisotropic nanostructures is governed by either oriented attachment or coalescence.^67−69^ Oriented attachment produces anisotropic regular nanostructures with no defects, while coalescence produces anisotropic irregular nanostructures with abundant defects. We believe that the mechanism of coalescence accounts for the formation of the anisotropic, irregular nanoparticles (right-bottom panel in Figure 10_iv_).

Moreover, adding a high content of methanol also favors the formation of [PbBr_4_]^2–^ complexes in addition to the formation of CsBrPb_3_. The [PbBr_4_]^2–^ complexes formed through a combination between [PbBr_3_]^−^ complexes and Br^–^ anions that were released from CsBr dissociation. Different from [PbBr_3_]^−^ complexes, [PbBr_4_]^2–^ complexes cannot be encapsulated by P2VP cores and preferentially remain outside of micelles through methanol solvation. This is because there is strong electrostatic repulsion between lone pairs of electrons in the pyridine and [PbBr_4_]^2–^ complexes.

Furthermore, [PbBr_3_]^−^ and [PbBr_4_]^2–^ complexes are still reactive and can bind with CsPbBr_3_ monoclinic lattices to form CsPb_2_Br_5_ tetragonal lattices through Ostwald ripening (Figure 10_v_). This reason explains that when the micellization power of PS-*b*-P2VP is reduced at a high content of methanol, the CsPbBr_3_ monoclinic lattices cannot be stabilized by micelles. Fused micelles or aggregated micelles also provide an effective pathway for transformation to CsPb_2_Br_5_ tetragonal crystals from CsPbBr_3_ monoclinic crystals. The subsequent growth of CsPb_2_Br_5_ tetragonal crystals produces CsPb_2_Br_5_ nanoplates with large lateral dimensions. Because of the large lateral dimensions, the nanoplates cannot be encapsulated by individual micelles but are adsorbed by a collection of micelles instead. Nevertheless, when the dimension of the CsPb_2_Br_5_ nanoplates is beyond a critical threshold, Brownian motion cannot offer a sufficient driving force to stabilize the dispersion of the CsPb_2_Br_5_ nanoplates. Eventually, gravity dominates Brownian motion, causing CsPb_2_Br_5_ nanoplates to precipitate in the perovskite solutions.

## Conclusions

We have demonstrated how the BCP-templating approach using PS-*b*-P2VP micelles synthesizes CsPbBr_3_ crystals with good PL performance and long-term stability in solution. PS-*b*-P2VP chains form spherical micelles in toluene, where PS blocks form shells and P2VP blocks form cores. We found that P2VP chains bearing lone pairs of electrons can induce the dissociation and complexation of PbBr_2_ crystals to form [PbBr_3_]^−^ complexes through a combination of micro- and nanoemulsion (i.e., multiple emulsion). The microemulsion is imparted by the steric repulsion of swelling PS chains and by the adsorption of P2VP chains onto PbBr_2_ microcrystals. The nanoemulsion relies on the capture of [PbBr_3_]^−^ complexes within P2VP cores through coordination reactions. Nevertheless, prolonged stirring is necessary to increase the content of [PbBr_3_]^−^ complexes captured within P2VP cores due to slow dissociation, complexation, and coordination reactions in toluene.

Considering that toluene is a nonsolvent for CsBr, CsBr salts must be separately dissolved in methanol to prepare CsBr/methanol solutions. Using neutral methanol to produce solvated Cs^+^ and Br^–^ ions results in two benefits. First, methanol is miscible with toluene. The solvated Cs^+^ and Br^–^ ions would experience a low enthalpic penalty with toluene. Second, methanol can increase the diffusion of Cs^+^ cations into P2VP cores and accelerate their binding with [PbBr_3_]^−^ complexes. Nevertheless, the methanol content should be carefully optimized. The SAXS and WAXD data demonstrate that a high content of added methanol reduces the micellization of PS-*b*-P2VP in toluene and increases the formation of [PbBr_4_]^2–^ complexes. At a high methanol content, a transformation from CsPbBr_3_ nanocrystals to CsPb_2_Br_5_ microcrystals through Ostwald ripening cannot be prevented by PS-*b*-P2VP micelles. Thus, CsPbBr_3_ nanocrystals synthesized at a high methanol content have low PL stability and performance.

Due to the fully inorganic nature of CsBr, it has limited solubility in methanol (up to 15 mg/mL) and cannot dissolve in toluene. Consequently, the stoichiometric imbalance between high PbBr_2_ and low CsBr concentrations results in the formation of small CsPbBr_3_ nanoparticles. Under these conditions, CsPb_2_Br_5_ nanoplates are more stable and tend to form over time, transforming CsPbBr_3_ nanoparticles into CsPb_2_Br_5_ nanoplates during prolonged storage. To address this limitation, we plan to use methylammonium bromide (MABr) as a replacement for CsBr, as MABr is highly soluble in methanol due to the organic nature of MA^+^ cations. We expect that a higher concentration of MABr will promote the formation of stable MAPbBr_3_ nanoparticles with well-defined shapes and sizes. Given that the addition of methanol reduces PS-*b*-P2VP micellization in toluene, we plan to use *o*-xylene or 1,3,5-trimethylbenzene as alternative solvents. PS-*b*-P2VP micellization is stronger in these solvents compared with toluene, potentially improving the encapsulation efficiency. Besides, future work will focus on quantifying product yield in the C-set and E-set solutions using thermogravimetric analysis (TGA) combined with direct pyrolysis mass spectrometry, which can differentiate between precursors (CsBr and PbBr_2_) and PS-*b*-P2VP based on their decomposition temperatures. Additionally, to improve the purity of encapsulated nanoparticles, selective precipitation techniques using poor solvents are being explored. We are also optimizing the content of PS-*b*-P2VP micelles as reducing the number of empty micelles could enhance the encapsulation efficiency. These improvements will be addressed in future studies.

## Supporting Information Available

Photos of precursor solutions with and without PS-*b*-P2VP in toluene (Figure S1); quantitative analysis of the TEM image of core–shell micelles (Figure S2); HR-TEM image of P2VP cores with [PbBr_3_]^−^ complexes (Figure S3); EDS spectrum and TEM image of a selected PbBr_2_ microcrystal (Figure S4); TEM image of cluster micelles (Figure S5); photos of the E-set solutions during various durations of storage at room temperature (Figure S6); optical properties of additional E-set solutions (Figure S7); PLQY and lifetime of additional E-set solutions (Table S1); TEM images and EDS profiles (Figure S8); XRD profiles of precipitates (Figure S9); UV–vis absorbance spectra of supernatants (Figure S10); UV–vis absorbance spectra of PbBr_2_ and its mixture with CsBr in toluene, with or without the addition of homopolymers (Figure S11); effects of stirring rates on PbBr_2_ complexation (Figure S12); temperature effects on PbBr_2_ complexation, micellar aggregation, and CsPbBr_3_ formation (Figures S13 and S14); synthesis of CsPbBr_3_ by directly mixing PbBr_2_ and CsBr in toluene (Figure S15); solvent effects on CsBr dissolution and CsPbBr_3_ formation (Figures S16–18); litmus-paper tests (Figure S19) (PDF)
