# Supplementary material for: Tuning Perovskite Nanocrystal Synthesis via Amphiphilic Block Copolymer Templates and Solvent Interactions
Source: ACS Appl Mater Interfaces. 2024 Oct 30;16(45):62664–79. doi: 10.1021/acsami.4c13822 (PMC11565575; doi:10.1021/acsami.4c13822)
Supplement: Supplementary file 1 — am4c13822_si_001.pdf [file am4c13822_si_001.pdf]

Supporting Information

Tuning Perovskite Nanocrystals Synthesis via Amphiphilic Block Copolymer Templates and Solvent Interactions

Ya-Sen Sun[1]*, Kuan-Wei Wu[1] and Orion Shih[2]

1. *Department of Chemical Engineering, National Cheng Kung University, Tainan 701, Taiwan*
2. *National Synchrotron Radiation Research Center, Hsinchu 30076, Taiwan*

*corresponding author: Y. S. Sun (Email: yssun@gs.ncku.edu.tw)

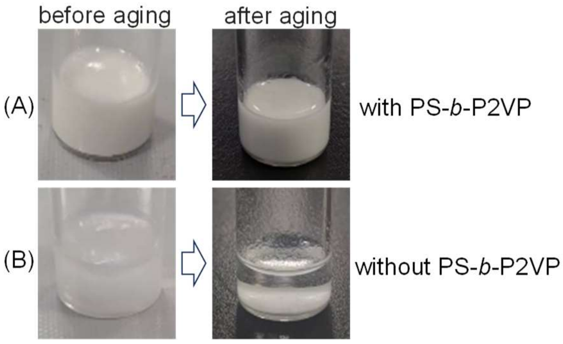

Figure S1. Photos of two solutions that contain $PbBr_2$ at 50 mg/mL in toluene (A) with and (B) without PS-*b*-P2VP (5mg/mL). No centrifugation was performed on the two solutions.

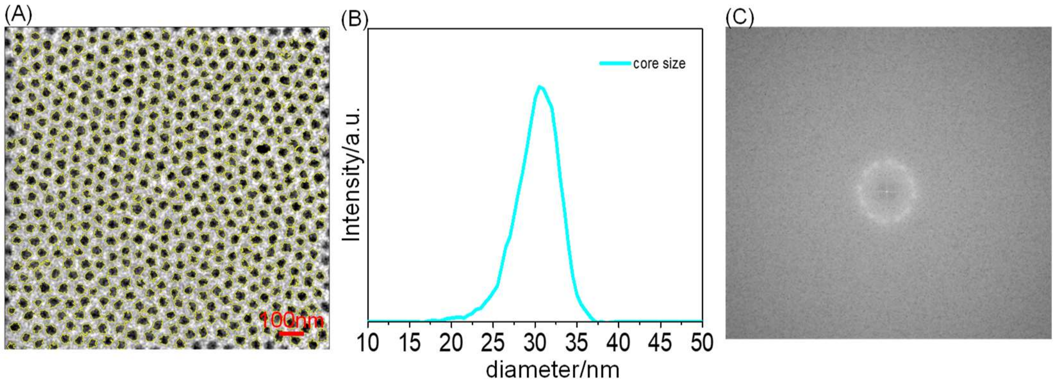

Figure S2. Quantitative analysis of (A, B) the P2VP core and (C) Fast Fourier Transformation (FFT) for Figure 4A. The FFT was performed using the ImageJ software, which allowed us to measure the periodicity of the P2VP domains in the block copolymer micelles.

Figure S2C show a diffuse ring, arising from the inter-micelle correlation of the micelles. The spatial frequency (i.e., the radius of the diffuse ring) obtained from the

FFT pattern was then converted to the corresponding real-space distance, giving the inter-core distance of approximately 45 nm.

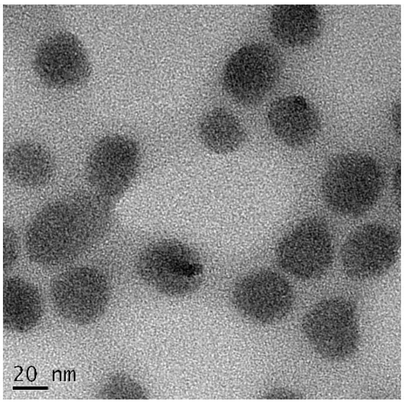

Figure S3. High-magnification TEM image of micelles enriched with $[PbBr_3]^-$ complexes.

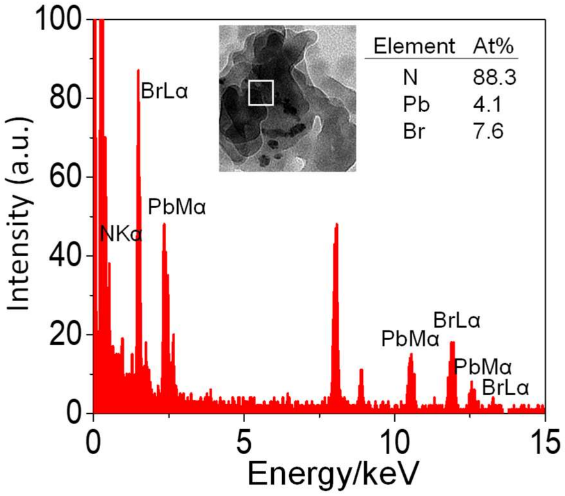

Figure S4. EDS spectrum of a selected $PbBr_2$ microcrystal. The atomic ratio of Pb to Br is approximately 1:2. The inset shows the TEM image of the $PbBr_2$ microcrystal selected for EDS analysis.

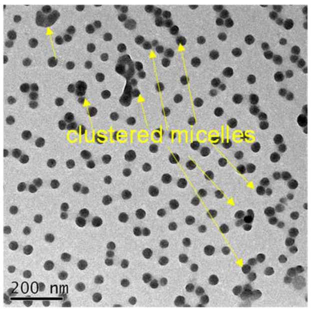

Figure S5. TEM image of clustered micelles, coexisting with individual micelles.

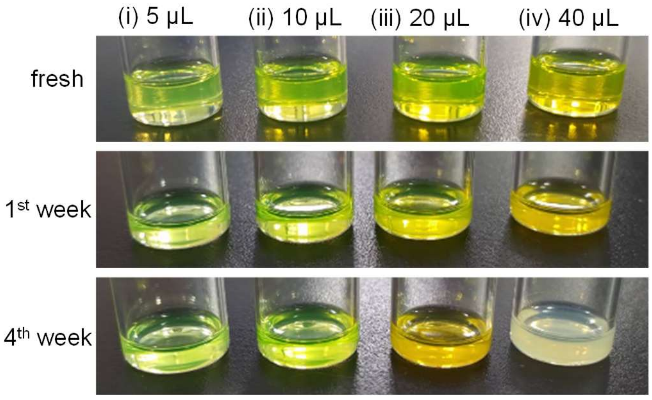

Figure S6. Photos measured for the E-set solutions discussed in Figure 5A during storage of various periods of time at ambient conditions without controlled humidity.

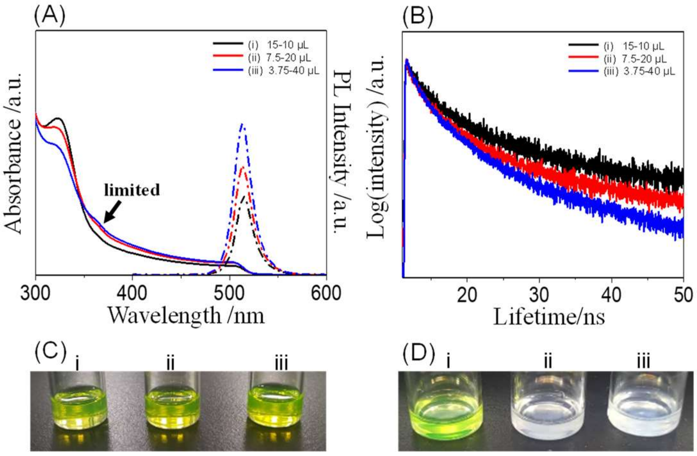

Figure S7. (A) UV-vis absorbance/PL spectra, (B) TRPL spectra and (C) photos of E-set solutions that were fleshly prepared by adding different aliquots of these solutions to the precursor supernatants, maintaining a consistent CsBr content: (i) 10 µL for 15 mg/mL, (ii) 20 µL for 7.5 mg/mL, and (iii) 40 µL for 3.75 mg/mL. (D) Photos of the E-set solutions taken after prolonged storage at room temperature for four months without controlled humidity.

Table S1. PLQY and average lifetime of the freshly prepared E-set solutions of Figure S7.

CsBr in MeOH (mg/mL)	MeOH (μL)	PLQY (%)	average lifetime (ns)
15	10	84.46	14.32
7.5	20	67.11	10.4
3.75	40	53.40	9.82

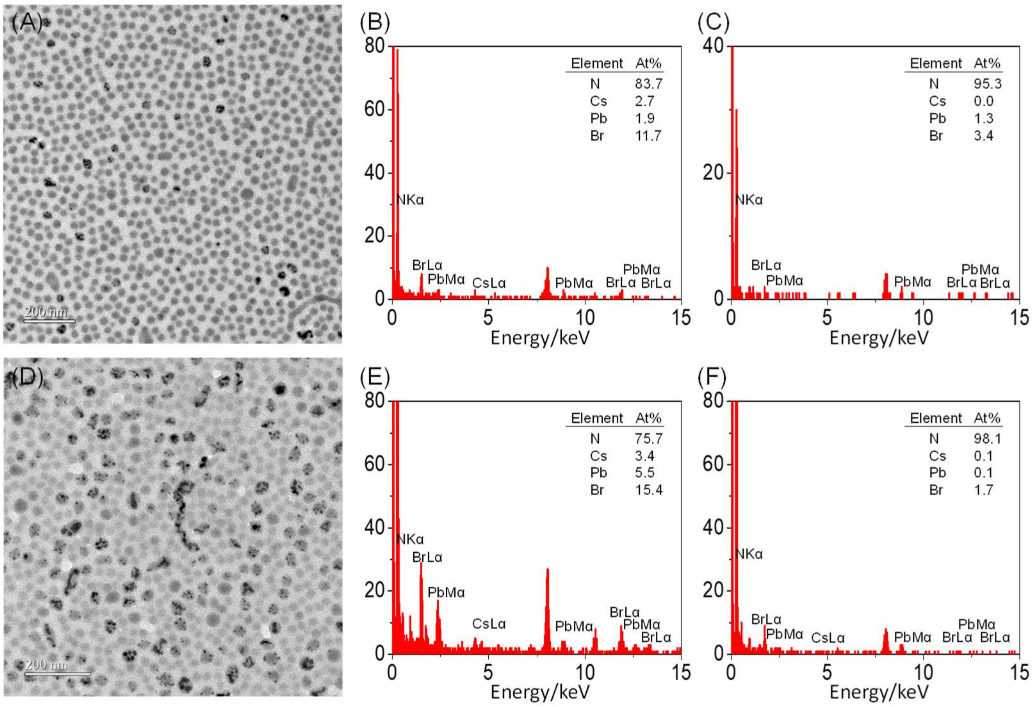

Figure S8. (A, D) low- and high-magnification TEM images, and (B-C, E-F) EDS profiles. The data were measured on a dried state of two fresh E-set solutions with addition of (A-C) 5 and (D-F) 20 μL of a D-set solution into C-set solutions. EDS spectra (B, E) were collected on selected micelle-encapsulated $CsPbBr_3$ nanoparticles while EDS spectra (C, F) were collected on selected nanoparticle-free micelles.

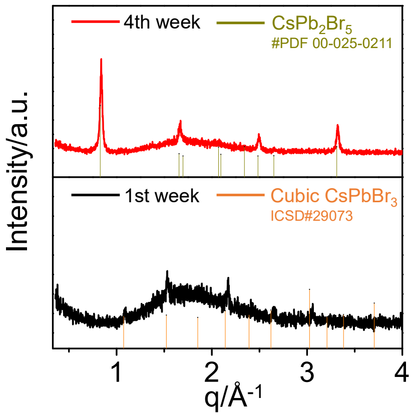

Figure S9. WAXD profiles of reprecipitates forming during storage for various times in the E-set solution discussed in Figure $S6_{iv}$. The precipitates were collected by centrifugation at 8000 rpm (5 min) from an E-set solution after one and four weeks of storage. The WAXD profiles were measured at a scan rate of 10°/min by in-house X-ray (Rigaku Ultima IV-9407F701) of $\lambda_{Cu,\,k\alpha}$=1.5418 Å, performed at voltage=40 V and current=20 mA.

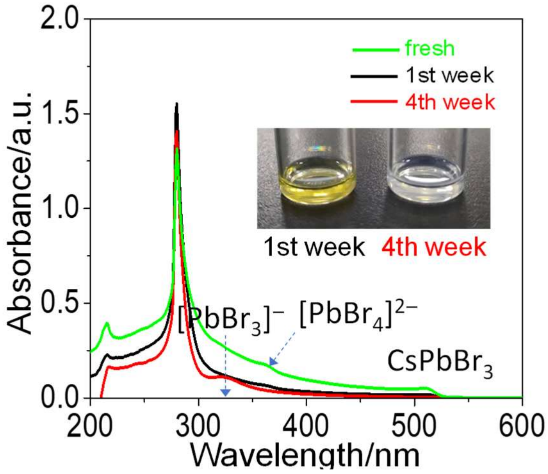

Figure S10. UV-vis absorbance spectra of supernatants obtained by centrifugation (8000 rpm, 5 min) on the E-set solution discussed in Figure $S6_{iv}$ after one- and four-week storage. The UV-vis absorbance spectrum of a fresh supernatant is shown together for a comparison.

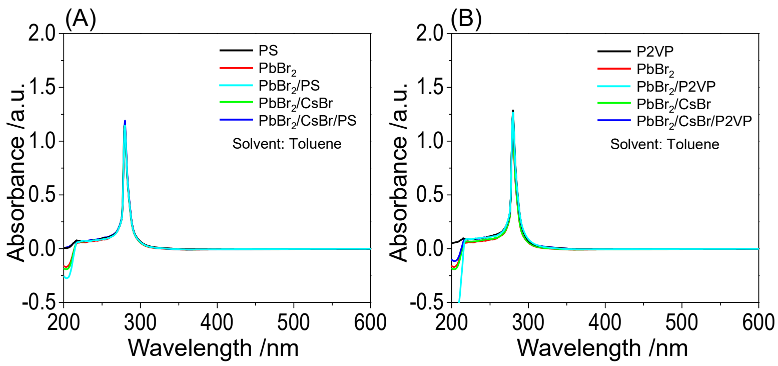

Figure S11. UV-Vis absorbance spectra of $PbBr_2$ and its mixture with CsBr in toluene, with or without the addition of (A) PS or (B) P2VP.

Effects of Stirring Rates on $PbBr_2$ Complexation.

In additional to stirring time, we also tested stirring speeds on the kinetics of $PbBr_2$ complexation at room temperature. Figure S12 demonstrates that no stirring cannot effectively result in $PbBr_2$ complexation. The absence of the absorbance band of $[PbBr_3]^-$ complexes indicates that the adsorption of PS-b-P2VP micelles onto the surface of $PbBr_2$ microparticles would take longer time in the absence of stirring. On the other hand, a high rate of stirring at room temperature cannot further enhance maximum $PbBr_2$ complexation.

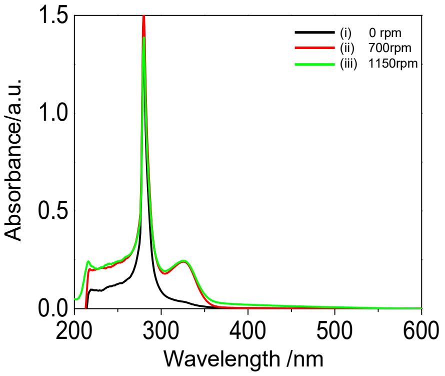

Figure S12. UV-Vis absorbance spectra of C-set solutions that were prepared by stirring at 0, 700 and 1150 rpm. The stirring was performed at 25 °C for 48 hours. Before measuring UV-vis absorbance spectra, the C-set solutions were centrifugated at 1000 rpm for 1 min.

Temperature Effects on $PbBr_2$ Complexation and Micellar Aggregates.

We also tested 45 and 70 ºC to study effects of stirring temperatures on $PbBr_2$ complexation and $CsPbBr_3$ formation. Although stirring at 45 and 70 ºC can effectively increase $PbBr_2$ complexation, the high-temperature stirring also significantly lead to inter-micelle clustering (Figure S13). An increased clustering inversely prohibited the formation of $CsPbBr_3$ nanoparticles with superior PL properties (Figure S14). These tests justify why stirring at 700 rpm at 25 ºC for hierarchical emulsion and $PbBr_2$ complexation is critical.

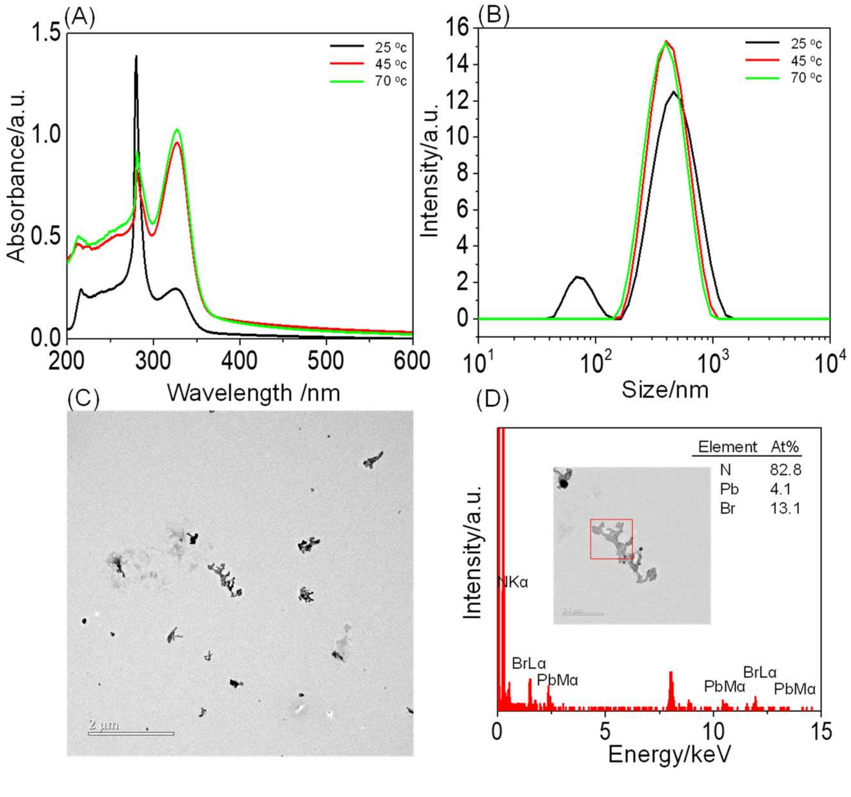

Figure S13. (A) UV-vis absorbance spectra and (B) DLS spectra of C-set solutions that were prepared by stirring at (i) 25, (ii) 45 and (iii) 70 ºC for 48 hours followed by centrifugation at 1000 rpm (1 min). (C) TEM image and (D) EDS 1D profile collected in a dried state for the C-set solution prepared at 70 ºC. Inset in (D) represents the image of an aggregate selected for measuring the ESD profile.

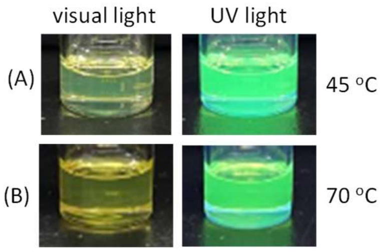

Figure S14. Photos of E-set solutions after centrifugation at 8000 rpm for 5 minutes. The E-set solutions were prepared using the C-set solutions, which had been stirred at (A) 45 °C and (B) 70 °C for 48 hours, followed by an initial centrifugation at 1000 rpm for 1 minute. The top solution exhibits a PLQY of 29.7%, while the bottom solution shows a PLQY of 6.32% under excitation at λ = 375 nm.

Synthesis of $CsPbBr_3$ by Directly Mixing $PbBr_2$ and CsBr in Toluene.

Another control experiment was conducted by simultaneously adding CsBr (30 mg) and $PbBr_2$ (50 mg) in the presence of PS-b-P2VP in toluene, followed by the addition of 10 μL methanol. The solution was stirred at 700 rpm at room temperature and centrifugated at 8000 rpm for 5 minutes. However, this approach did not yield encapsulated $CsPbBr_3$ nanoparticles with good PL (Figure S15). The reason is that CsBr directly interacted with $PbBr_2$ microparticles, thus forming $CsPbBr_3$ microparticles, which were significantly larger than the dimensions of the P2VP cores. As a result, PS-b-P2VP micelles exhibited poor encapsulation efficiency for the $CsPbBr_3$ microparticles. Additionally, the formation of $CsPbBr_3$ microparticles interfered with and dominated the $PbBr_2$ complexation within the P2VP cores, as the $PbBr_2$ complexation process occurs more slowly (taking several hours to days) compared to the rapid formation of $CsPbBr_3$ microparticles (within seconds to minutes). This control experiment highlights the importance of $PbBr_2$ complexation into $[PbBr_3]^-$ complexes encapsulated within the P2VP cores for the successful formation of encapsulated $CsPbBr_3$ nanoparticles.

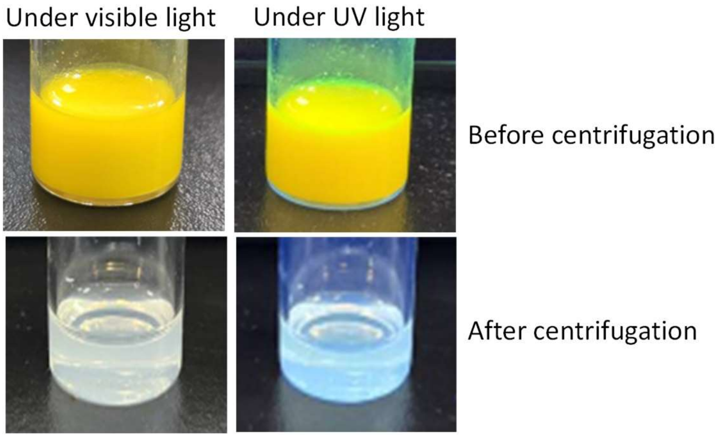

Figure S15. Photos of additional E-set solutions before and after centrifugation at 8000 rpm 5 min. The E-set solutions were prepared by simultaneously adding CsBr (30 mg) and $PbBr_2$ (50 mg) in the presence of PS-*b*-P2VP in toluene, followed by the addition of 10 μL methanol. The solution was stirred at 700 rpm for 48 hours at room temperature and centrifugated at 8000 rpm for 5 minutes.

Solvent Effects on CsBr Dissolution and $CsPbBr_3$ Formation

Three solvents were tested for dissolving CsBr: dimethylformamide (DMF), methanol, and acetic acid (AC). Of these solvents, neutral methanol dissolved CsBr effectively at a concentration of 15 mg/mL. In contrast, CsBr exhibits poor solubility and thus exists as precipitates in both DMF and acetic acid (Figure S16).

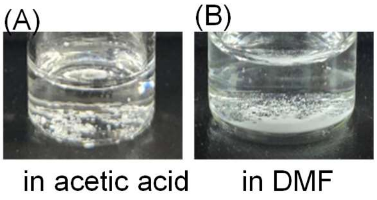

Figure S16. Photos of additional D-set solutions that were prepared by dissolving 15 mg CsBr in (A) acetic acid and (B) DMF.

Due to this limited solubility, $CsPbBr_3$ nanoparticles with superior PL could not be formed by adding CsBr aliquots that had been pre-dissolved in DMF, acetic acid, and a mixture of methanol and acetic acid (Figures S17 and S18).

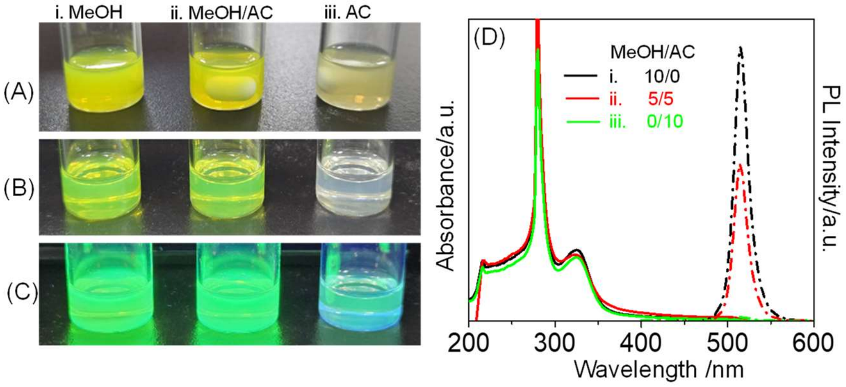

Figure S17. (A-C) Photos and (D) UV-vis absorbance/PL spectra of E-set solutions, for which CsBr solutions were prepared by using (i) methanol (MeOH), (ii) methanol/acetic acid mixture with equal volumes and (iii) acetic acid (AC). Photos (A) were taken under visual light before centrifugation. Photos (B) were taken under visual light after centrifugation. Photos (C) were taken under UV light after centrifugation. The solution ii shows a PLQY of 63.47% while the solution iii shows a PLQY of 13.84%.

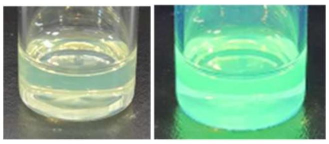

Figure S18. Photos of an E-set solution after centrifugation at 8000 rpm for 5 min. The E-set solution was prepared by adding a 10 μL CsBr/DMF aliquot into a centrifugated C-set solution. The solution shows a PLQY of 24.59%.

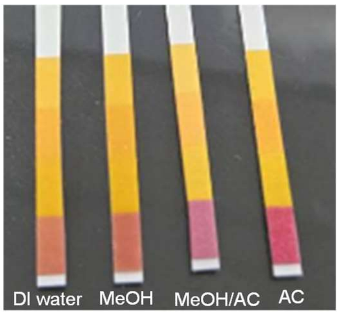

Figure S19. Litmus-paper tests on deionized water, methanol, methanol/acetic acid mixture and acetic acid.
